# On the Role of Bilateral Brain Hypofunction and Abnormal Lateralization of Cortical Information Flow as Neural Underpinnings of Conventional Metaphor Processing Impairment in Schizophrenia: An fMRI and EEG Study

**DOI:** 10.1007/s10548-021-00849-x

**Published:** 2021-05-10

**Authors:** Przemysław Adamczyk, Martin Jáni, Tomasz S. Ligeza, Olga Płonka, Piotr Błądziński, Miroslaw Wyczesany

**Affiliations:** 1 Institute of Psychology, Jagiellonian University, Ingardena 6, 30-060 Kraków, Poland; 2 Department of Psychiatry, Faculty of Medicine, Masaryk University and University Hospital Brno, Brno, Czech Republic; 3 Community Psychiatry and Psychosis Research Center, Chair of Psychiatry, Medical College, Jagiellonian University, Kraków, Poland

**Keywords:** Metaphor, Schizophrenia, Effective connectivity, Lateralization, Electroencephalography, Directed transfer function, Functional magnetic resonance imaging

## Abstract

Figurative language processing (e.g. metaphors) is commonly impaired in schizophrenia. In the present study, we investigated the neural activity and propagation of information within neural circuits related to the figurative speech, as a neural substrate of impaired conventional metaphor processing in schizophrenia. The study included 30 schizophrenia outpatients and 30 healthy controls, all of whom were assessed with a functional Magnetic Resonance Imaging (fMRI) and electroencephalography (EEG) punchline-based metaphor comprehension task including literal (neutral), figurative (metaphorical) and nonsense (absurd) endings. The blood oxygenation level-dependent signal was recorded with 3T MRI scanner and direction and strength of cortical information flow in the time course of task processing was estimated with a 64-channel EEG input for directed transfer function. The presented results revealed that the behavioral manifestation of impaired figurative language in schizophrenia is related to the hypofunction in the bilateral fronto-temporo-parietal brain regions (fMRI) and various differences in effective connectivity in the fronto-temporo-parietal circuit (EEG). Schizophrenia outpatients showed an abnormal pattern of connectivity during metaphor processing which was related to bilateral (but more pronounced at the left hemisphere) hypoactivation of the brain. Moreover, we found reversed lateralization patterns, i.e. a rightward-shifted pattern during metaphor processing in schizophrenia compared to the control group. In conclusion, the presented findings revealed that the impairment of the conventional metaphor processing in schizophrenia is related to the bilateral brain hypofunction, which supports the evidence on reversed lateralization of the language neural network and the existence of compensatory recruitment of alternative neural circuits in schizophrenia.

**Supplementary Information:**

The online version contains supplementary material available at 10.1007/s10548-021-00849-x.

## Introduction

Figurative speech, such as humor, metaphor, irony, or sarcasm, is an important element of pragmatic communication skills in everyday language. Such non-literal expressions interpreted literally are seemingly meaningless since they are incongruent with the actual situational context and reinterpretation of their semantic meaning is needed. The understanding of the figurative language requires the semantic shift between literal and non-literal meanings, to obtain full context coherence, allowing for the comprehensive elaboration of the intended meaning. Considering the neural basis for figurative stimuli processing in the healthy brain, the bilateral but left dominant neural network engagement of fronto-temporo-parietal brain regions has to be emphasized (for review see: Bohrn et al. 2012; Rapp et al. 2012; Reyes-Aguilar et al. 2018).

Figurative language processing as a specific communication skill is commonly impaired in schizophrenia (Adamczyk et al. 2016; Bozikas et al. 2007; Chakrabarty et al. 2014; Kircher et al. 2007; Mo et al. 2008; Mossaheb et al. 2014; Polimeni et al. 2010; Rapp et al. 2013; 2018; Varga et al. 2013). Literature data indicates that the principal causes of figurative language impairment are deficiencies in semantic cognition, i.e. set-shifting between literal and non-literal meanings in a given linguistic context and the general tendency to use concretism and literal language (Kircher et al. 2007; Kuperberg 2010; Kuperberg et al. 1998; Polimeni et al. 2010; Rapp et al. 2013, but see Elvevåg et al. 2011).

Besides clear-cut behavioral evidence of figurative language disturbances, the data regarding neural substrates of this deficit are only fragmentary and not sufficient to fully understand this phenomenon. In brief, functional Magnetic Resonance Imaging (fMRI) studies on humor (Adamczyk et al. 2017, 2018; Berger et al. 2018) and irony (Rapp et al. 2013; Varga et al. 2013) have revealed aberrant activity within the left frontal and right temporo-parietal cortices in schizophrenia. Moreover, reversed lateralization of information flow assessed with electroencephalography (EEG) has been found during humor processing (Adamczyk et al. 2019). Studies on metaphor processing in schizophrenia have revealed hyperactivation in the left inferior frontal gyrus (IFG) and suppressed activation in the right posterior temporal lobe (pTL) and the precuneus (Kircher et al. 2007, but see Mashal et al. 2014), or suppressed activation in the right IFG together with compensatory hyperactivation in the left fronto-parietal regions (Mashal et al. 2013, 2014). Next, simultaneous functional near-infrared spectroscopy (fNIRS) and EEG event-related potentials (ERPs) study have revealed abnormal left hemisphere (LH) activation related to impairments in metaphor processing, and non-specific alterations in N400 amplitude (Iakimova et al. 2005; Schneider et al. 2015). Lastly, the findings of a magnetoencephalographic (MEG) study (Zeev-Wolf et al. 2015) revealed enhanced brain activation in the right hemisphere (RH) at the early stage (170 ms) of metaphor processing and reduced bilateral brain activity at the later stage (350 ms). Overall, these findings suggest the existence of distributed functional disturbances in the fronto-temporo-parietal neural network during figurative speech processing in schizophrenia.

The interpretation of existing evidence in terms of abnormal lateralization of language processes in schizophrenia may allow for better organization and understanding of their mechanisms (Crow 2004; Leroux et al. 2015; Mitchell and Crow 2005). Currently, two complementarily intertwined neuro-linguistic theories of step-by-step figurative semantic processing are still under debate (Rapp et al. 2018). The coarse semantic coding theory (Beeman et al. 1994) assumes bi-hemispheric cooperation reflected in parallel and dichotomous streams of processing in the LH (literal/close semantic relationships) and RH (figurative/coarse/distant semantic concepts) (Beeman and Bowden 2000; Beeman et al. 1994, 2000; Jung-Beeman 2005). The most recent evidence indicates that the LH is associated with both literal and non-literal language processing, with enhanced activation of the RH especially visible during novel/unfamiliar figurative meaning processing (Diaz and Eppes 2018; Kavé et al. 2014; Kircher et al. 2007; Mashal and Faust 2008, 2009). This provides support for descendant theory, the graded salience hypothesis (GSH) (Giora 1997), which implicates bilateral but more excessive RH engagement during more distant (non-salient) semantic concepts, e.g. novel metaphor processing. The figurative meaning of commonly used conventional metaphors may be considered a more salient interpretation related to the primal LH engagement (Giora 1997, 2007; Giora et al. 2000; Stringaris et al. 2006, 2007).

The current study is aimed at determining how schizophrenia individuals differ from healthy subjects during the processing of conventional metaphors. On the one hand, conventional metaphors represent the most commonly used figurative expression in daily life, which makes them essential for pragmatic communication skills. On the other, it may be considered as the representative of the salient figurative meaning which stays in contrast to the non-salient figurative meanings, such as humor. Therefore, we intended to interpret these results in the GSH framework. Moreover, using a sophisticated effective connectivity methodology we wanted to step beyond classic functional data and examine the abnormalities of neural communication within the bilateral fronto-temporo-parietal circuit. Additionally, we wanted to compare current data with our previous results on humor comprehension (Adamczyk et al. 2019). In particular, the specificity of neural network disturbances in schizophrenia during processing of different aspects of figurative language, i.e. conventional metaphors (salient content) vs humorous jokes (non-salient content; Adamczyk et al. 2017, 2019). Noteworthy, the GSH predictions on the gradable hemispheric engagement are coherent with our previous findings on attenuated brain activity and shifted lateralization of information flow during humor (non-salient content of jokes) processing, i.e. schizophrenia outpatients were characterized by impairment related to the diminished activity of the left frontal and right parietal regions along to reversed leftward-shifted pattern of effective connectivity, compared to controls (Adamczyk et al. 2017, 2019). Thus, in the light of GSH predictions, we may expect that in the case of conventional metaphors (figurative but salient content) impairment will be related especially to the diminished activity of the LH regions (e.g. IFG, Precuneus, IPL) and we may expect reversed directionality of information flow compared to the controls, as a reflection of reversed language lateralization in schizophrenia (Mitchell and Crow 2005).

## Material and Methods

### Participants

The study included 30 schizophrenia outpatients and 30 healthy controls matched in age, sex, and education (Table 1). All subjects gave informed consent to participate in the experimental procedures: interview, Montreal Cognitive Assessment (MoCA; Nasreddine et al. 2005), fMRI scanning, and EEG recordings with additional assessment in the clinical group with the Positive and Negative Syndrome Scale (PANSS; Gaag et al. 2006; Kay et al. 1987) and the Brief Negative Symptom Scale (BNSS; Kirkpatrick et al. 2011). Exclusion criteria included a history of head injuries, seizures, substance dependence, or any current somatic illnesses. All clinical subjects were in a stable psychopathological condition before the experiment. All clinical subjects were taking antipsychotic medication, including conventional (1st generation: flupentixol, haloperidol, promazine) and/or atypical (2nd generation: amisulpride, clozapine, olanzapine, risperidone, sulpiride, quetiapine; aripiprazole) neuroleptics. Additionally, 4 patients received antidepressants (escitalopram, paroxetine), 11 anxiolytics (hydroxyzine), and 6 mood stabilizers (carbamazepine, lithium, valproic acid). All but two were right-handed, and all were native Polish speakers. All participants were remunerated (n = 60; €40). Procedures were designed in accordance with the ethical standards of the World Medical Association Declaration of Helsinki (2013) and approved by the Research Ethics Committee at the Institute of Psychology, Jagiellonian University, Krakow, Poland.

**Table 1:** Demographic and clinical data

Demographic and clinical data	Schizophrenia outpatients (n = 30)	Healthy controls (n = 30)	Test for between-group differences
*Demographic*	Mean (SD)	Mean (SD)	
Age	41.88 (8.83)	41.80 (8.68)	t = 0.72; ns
Sex: male/female	14/16	14/16	Chi^2^ = 0.000; ns
Education (in years)	15.22 (2.95)	16.30 (2.91)	t = 3.03; p < 0.005
MoCA	25.10 (3.64)	27.03 (1.96)	t = − 4.841; p < 0.01
*Clinical*	n (%)		
Schizophrenia diagnosis (ICD-10)			
Paranoid (F20.0)	27 (91%)		
Undifferentiated (F20.3)	2 (6%)		
Schizoaffective disorder (F25.0)	1 (3%)		
Type of pharmacotherapy:			
Typical anipsychotics	1 (3%)		
Atypical antipsychotics	27 (91%)		
Typical and atypical antipsychotics mixed	2 (6%)		
Anxiolytics	11 (37%)		
Antidepressants	4 (14%)		
Mood stabilizers	6 (20%)		
Characteristic of the illness	Mean (SD)		
Duration of psychosis (in years)	17.2 (8.57)		
Number of relapses	8.83 (7.33)		
Number of hospitalizations	8.6 (5.57)		
Chlorpromazine equivalent (mg/day)	425.33 (277.74)		
*PANSS*	Mean (SD)		
Total	61.23 (16.01)		
Positive	11.3 (4.15)		
Negative	16.9 (6.4)		
Disorganization	9.53 (3.95)		
Excitement	6.07 (2.24)		
Emotional distress	9.17 (3.17)		
*BNSS*			
Total	22.03 (13.54)		
Anhedonia	5.3 (3.98)		
Asociality	3.3 (2.38)		
Avolition	3.23 (2.36)		
Blunted effect	6.3 (4.51)		
Alogia	2.97 (2.51)		

Subjects demographics and clinical data were presented as n (%) for nominal variable and as mean (SD) for quantitative data. The significance level in all statistical analyses equalled alpha = 0.05

### Experimental Procedure

The experimental procedures were adapted from these described in our previous report on humor (Adamczyk et al. 2019). All substantial modifications for investigation on conventional metaphor processing are provided below. In two complementary MRI and EEG experiments, we used a punchline-based metaphor comprehension task. It contained short stories with three possible endings/punchlines: neutral (NEU)—literal meaning, absurd (ABS)—meaningless sentence, and metaphorical (MET)—figurative meaning. The experimental stimuli of the punchline-based metaphor comprehension task are consistent with other commonly used experimental approaches to metaphor processing (Bohrn et al. 2012; Iakimova et al. 2005; Rapp et al. 2012; Reyes-Aguilar et al. 2018; Rossetti et al. 2018; Schneider et al. 2015). Additionally, for comparison purposes, we adapted the previously used experimental model related to the step-by-step theory of humor (Chan et al. 2013; Suls 1972; Wyer and Collins 1992), that includes the following stages: 1. incongruity detection (recognition of semantic conflict between the literal meaning of punchline within story context), 2. incongruity resolution (semantic-shift of punchline meaning coherent with context), and 3. elaboration (higher-order cognitive inference, self-referential thoughts and/or emotional reaction). The adapted model and assessed contrast reflect 1. incongruity detection and absurd processing (ABS vs NEU; semantic conflict of non-salient but not metaphorical stimuli), 2. metaphor recognition and elaboration (MET vs ABS; comprehensible and metaphorical stimuli, but without incongruity detection element), and 3. complete metaphor processing (MET vs NEU), where all metaphorical components are present, but without elements of literal processing.

#### Punchline-Based Metaphor Comprehension Task

204 metaphorical phrases commonly used in the Polish language (i.e. conventional metaphors) were selected to prepare short stories (i.e. contextual situation of question–answer dialogues) with punchlines (i.e. answers) consisting of metaphorical sentences; they were then adapted to create three types of endings, i.e. NEU, ABS, MET following the punchline-based humor comprehension task procedure (Adamczyk et al. 2017, 2019). Next, 612 stories were divided into three separate sets (3 × 68 MET, 68 NEU, 68 ABS), thus ensuring that each person rated only one version of the same story/setup; they were then presented for pre-selective judgment to 60 healthy people (25 males, 35 females; mean age 27.65 ± 6.44 SD, min. 19–max. 48), who rated comprehensibility and metaphoricity for all conditions on a 1–9 Likert scale (from 1 = incomprehensible/non-metaphorical to 9 = comprehensible/metaphorical). The stories were presented on a computer screen and ratings were provided using a keyboard. Then, for proper test the selected stories were chosen only when they were rated > 8 on the comprehensibility and metaphoricity ratings for MET; > 8 for comprehensibility and < 2 on metaphoricity for NEU; < 2 on the comprehensibility and metaphoricity ratings for ABS conditions. For the proper test, we selected final sets of stimuli divided into two experimental procedures (i.e. for EEG and fMRI assessment), each consisting of 90 stories (30 MET, 30 NEU, 30 ABS). Each stimulus contained two components: a setup (6–21 words long; mean = 12.06; SD = 2.58) and a punchline (3–12 words long; mean = 6.73; SD = 1.80). Ratings are presented in Online Resource 1. The conditions did not differ in word count (t = − 1.01, p = 0.31). The examples of three conditions of stimuli/punchlines in English translation: (1) MET condition—Setup: ‘Man riding a bike accidentally hits a girl walking on the street.—I am sorry, are you all right? Punchline: ‘-No, I am sorry, I shouldn’t walk with my head in the clouds.’; (2) NEU condition—Setup: ‘Man is coming back home after an unusually long day at work.—Honey, why are you so late? The dinner is cold already. Punchline: ‘- I am very sorry. I had to finish an important project.’; (3) ABS condition—Setup: ‘Two colleagues are talking at work.—I can’t believe that John is earning at the same position more money than me!’ Punchline: ‘- The copy machine broke yesterday.’

#### Experimental Task

The punchline-based metaphor comprehension task was designed and presented using PsychoPy v1.82.01 software (Peirce 2009; Peirce et al. 2019). During an fMRI session, the stimuli were presented on an MRI compatible screen, and responses were collected using fiber-optic response button grips (Nordic Neuro Lab, Bergen, Norway) by the right- and left-hand index fingers (as yes and no, respectively); during an EEG session, the stimuli were presented on a computer screen and responses collected on a standard keyboard (left/right shift to select a yes/no rating, respectively). Before the proper test, a short instruction was presented, followed by a few examples to practice responding. Then, each participant was presented with 90 stories in a randomized order, with 30 items for each of the three endings (MET, NEU, ABS). The task started by displaying the word ‘start’ and fixation-cross. Presentation time was 8 s for setups and 5 s for punchlines. Then, the comprehensibility and metaphoricity rating scales appeared one-by-one. Dichotomous responses on subjective judgments of comprehensibility and metaphoricity were collected (left/right to select a yes/no rating, respectively) without any time restriction. The trials were separated by interstimulus intervals (ISI) randomly varying from 3 to 9 s. The session included breaks after the presentation of the 30th and 60th stimuli. The total time for each fMRI and EEG recording was approx. 1 h per person. All the subjects had the same order of experimental procedures, i.e. (i) interview, cognitive screening, and clinical assessment followed by (ii) fMRI scanning within max. two consecutive days, and complemented by (iii) EEG recording within max. two consecutive weeks.

### fMRI Data Acquisition and Preprocessing

Magnetic resonance imaging (MRI) was executed using a 3T scanner (Magnetom Skyra, Siemens) at Malopolska Centre of Biotechnology, Krakow, Poland. The acquisition was performed with a 64-channel head coil. High-resolution, anatomical images were acquired using the T1 MPRAGE sequence (sagittal slices; 1 × 1 ×  1.1 mm^3^ voxel size; TR = 1800 ms, TE = 2.37 ms). Functional images were acquired using the EPI sequence. The scan parameters were as follows: 3 mm isotropic voxel, TR = 2000 ms, TE = 27 ms, flip angle = 75°, FOV 192 × 192 mm^2^, GRAPPA acceleration factor 2, and phase encoding A/P. Whole-brain images were covered with 37 axial slices with a 20% gap between slices (distant factor = 0.6 mm), taken in an interleaved, ascending fashion. Additionally, a B0 inhomogeneity field map was acquired with a dual-echo gradient-echo sequence matched spatially with fMRI scans (TE1 = 4.92 ms, TE2 = 7.38 ms, TR = 466 ms).

Data processing was performed using the SPM12 software (Statistical Parametric Mapping, Wellcome Department of Cognitive Neurology, London, UK). The processing pipeline included the following steps: calculation of voxel displacement using FieldMap, unwarping through a field map correction, slice timing correction, motion correction (realignment) of functional images using a six-parameter rigid body transformation, co-registration to the anatomical reference image, segmentation into separate tissues (white matter, grey matter and cerebrospinal fluid), normalization to a standard MNI stereotaxic space with 3 mm isotropic voxels using a 12-parameter affine transformation and spatial smoothing using a 5 mm Gaussian kernel. One subject from the clinical group was eliminated from further analysis due to a scan rejection (i.e. excessive movements) rate greater than 20%. Low-frequency signal components were removed using a high-pass filter with a cutoff of 128 s.

### EEG Recording and Preprocessing

EEG recording was carried out using a Biosemi Active Two amplifier and 64 active electrodes placed on a standard 10–10 headcap with 256 Hz sample rate. Four additional sensors were used to record oculomotor activity, and two were used for offline linked mastoid reference. The preprocessing was performed using EEGlab toolbox (Delorme and Makeig, 2004): filtering (3 to 45 Hz with zero phase-shift filters), downsampling to 128 Hz, a custom procedure for removing blink contamination (subtracting individually fitted blink curve; Wyczesany et al. 2018), artifact rejection (the segments exceeding the 100 μV threshold on any of the electrodes were dropped; muscle artifacts were removed by rejecting the epochs with spectral power in a range 35–45 Hz and exceeding 30 dB over the average to identify muscle activity contamination in the signal) and epoching (time windows − 1 to 3 s relative to punchline onset. The model order was set to 8 according to the Akaike Information Criterion.

### Directed Transfer Function

To estimate effective (directional) connectivity the directed transfer function (DTF) method was used (Kamiński and Blinowska 1991). DTF is based on the multivariate autoregressive modeling (MVAR) and provides the estimation of causal relations between signals based on the Granger concept. It defines a signal Y as causal for a signal X if values of X can be better predicted using previous values of both signals X and Y than using previous values of signal X alone. As a multivariate method, it accounts for the whole set of signals at once. The detailed description of the method is beyond the scope of the article but can be found elsewhere (Blinowska 2011).

### Effective Connectivity Analysis

The DTF was calculated in a single MVAR model including all the channels of interest, i.e. the length of available data was then checked across each subject/condition to confirm the use of the 1 s window length in MVAR modeling, using the formula: W ≥ 10*(pk/N) where: W—required minimum window length in samples, p—model order, k—number of channels and N—total number of epochs within particular valence condition (Korzeniewska et al. 2003).

Based on the EEG montage brain atlas (Koessler et al. 2009), 29 electrodes were selected that corresponded to the regions of interest (ROIs) that were chosen based on the existing literature (Reyes-Aguilar et al. 2018) and previous findings on humor (Adamczyk et al. 2019): orbital prefrontal cortex (oPFC: Fpz), ventromedial PFC (vmPFC: Fp1/Fp2); dorsomedial PFC (dmPFC: AFz-Fz); dorsolateral PFC (dlPFC: AF3-AF7-F3/AF4-AF8-F4); inferior frontal gyrus (IFG: F7-FC5/F8-FC6); anterior temporal lobe (aTL: T7/T8); posterior TL (pTL: TP7-P7/TP8-P8); temporo-parietal junction (TPJ: CP5/CP6); inferior parietal lobule (IPL: P3–P5/P4–P6); precuneus (Prec: P1–P2).

To estimate effective connectivity the sensor-level approach was chosen, firstly to provide comparisons with our previous data on language deficits in schizophrenia (Adamczyk et al. 2019). Secondly, although the advantages and drawbacks of both source-level and sensor-level approaches are debated (Babiloni et al. 2020; Kaminski and Blinowska 2017; Mahjoory et al. 2017), it should be remembered that source-based connectivity is not a universal solution to the influence of volume conduction. The undetermination of the source localization problem causes some spatial inaccuracies and is always associated with a fuzziness of their reconstructions, leading to unwanted source leakage effects. This latter phenomenon can be responsible for the possible detection of spurious connectivity effects. On the other hand, the DTF method is based on autoregressive modeling, which does not take into account instantaneous connectivity (t = 0) but only the relationships shifted in time. This limits the influence of volume conduction.

Non-normalized DTF values for all pairs of electrodes selected to match our predefined ROIs were calculated in the beta band (14 Hz–25 Hz) for six-time bins of 1 s length with 0.5 s shift (from − 1 to 0 up to 2–3 s relative to punchline onset) using Multar software (Department of Biomedical Physics, University of Warsaw, Poland). The beta band was chosen for DTF analysis based on the previous work in the field which showed the most stable effects on tracing cortical connectivity related to experimental manipulation (Ferdek et al. 2019; Ligeza et al. 2021; Wyczesany et al. 2014), and considered to well reflect language processing (Scaltritti et al. 2020; Spironelli et al. 2020). This was also confirmed in our previous report on humor disturbances in schizophrenia (Adamczyk et al. 2019).

The connectivity value of the first pre-punchline bin (i.e. – 1 to 0 s) was subtracted from all the following bins (0–1 s, 0.5–1.5 s, 1–2 s, 1.5–2.5 s, 2–3 s) to obtain baseline-corrected DTF values. To control the quality of autoregressive model fitting, residual noise matrices were determined for all subjects. The distributions of the obtained DTF values were checked to identify and reject possible extremes, which were defined as falling below Q1 − 1.5*IQR or above Q3 + 1.5*IQR, where Q is quartile and IQR is interquartile range (Ligeza et al. 2016).

### Statistical Analysis

For behavioral data, at the first step, individual means for comprehensibility and metaphoricity ratings and reaction times were computed separately for each condition (MET, ABS, NEU) as the total sum from both procedures (EEG and fMRI). At the second step, for each condition, the conjunction ratings were computed for the factual level of correct responses and the detection of specific error types occurrence. The correct rating responses were: MET (comprehensible and metaphorical; yes/yes); NEU (comprehensible and non-metaphorical; yes/no); ABS (incomprehensible and non-metaphorical; no/no). Each other rating combination was assumed as an erroneous response. Between-group differences, due to the non-normal distribution of data were analyzed with the *U* Mann–Whitney test with alpha = 0.05.

For fMRI data, the general linear model (GLM) was applied in a canonical pattern of the hemodynamic function. The 1st level model included the setup, the punchline (with three levels: MET, ABS, NEU), and the response period. Additionally, two other models were used and included the same stages, but the punchline was not distinguished between the conditions and used as a silent regressor. Instead, the individual ratings on either comprehensibility or metaphoricity for each punchline were included, respectively. At the second level of analysis, based on previously assessed contrasts from humor experiments (Adamczyk et al. 2017) three main within-group and between-group contrasts were provided separately: ABS vs NEU, MET vs ABS, MET vs NEU. Additionally, to further differentiate the areas involved in processing the conventional metaphors processing, analysis of the within-group and between-group contrasts were performed using both subjective ratings separately (i.e. comprehensibility and metaphoricity). A non-parametric whole-brain voxel-wise Pseudo-*t*-test with variance smoothed with full-width-at-half-maximum (FWHM) 5 × 5 × 5 mm and 10,000 permutations using the Statistical NonParametric Mapping (SnPM13) toolbox (http://warwick.ac.uk/snpm) was used. Localizations were reported as a local maximum threshold with k ≥ 10 voxels threshold and with uncorrected alpha = 0.001.

For EEG data, the linear mixed models' statistics were computed using the lme4 R package (Bates et al. 2015) to test the effects of conditions and groups on connectivity between selected ROIs. A model with group (healthy controls, schizophrenia outpatients), condition (MET, NEU, ABS), ROI (in total 29 electrodes forming predefined regions), and time window (0–1 s, 0.5–1.5 s, 1–2 s, 1.5–2.5 s, 2–3 s) as fixed factors and subjects as a random factor were analyzed. In the case of within-group analysis, the group reference level was fixed to the respective group. For the between-group analysis, the interaction term between condition and group was considered. Bad channels were removed after the visual inspection supported by semiautomatic detection based on trimmed (5% tail removed) data statistics (including standard deviation, skewness, and kurtosis of signal distribution). In the case of the removed channel, missing data were introduced to the statistical model. Finally, effective connectivity maps were created to reveal the between-group differences in the information flow between specified ROIs of figurative language-related networks. Data were visualized using Trand3D 1.2 software (Department of Biomedical Physics, University of Warsaw; Blinowski et al. 2014) in three pairs of directional contrasts, based on previously assessed contrasts from humor experiments (Adamczyk et al. 2017, 2019; Chan et al. 2012, 2013): ABS vs NEU, MET vs ABS, MET vs NEU. Effective connectivity maps were created to reveal the between-group differences in the information flow. To decrease the risk of false positives due to multiple comparisons, FDR correction was set to alpha at 0.01.

## Results

Clinical subjects were less accurate in comprehensibility and metaphoricity ratings on the punchline-based metaphor comprehension task compared to healthy controls (Table 2). Results of comprehensibility ratings in schizophrenia showed difficulties with understanding all types of punchlines. In particular, the NEU and MET endings were rated as less understandable, while the ABS ones were rated as more comprehensible compared to healthy controls. Similarly, the metaphoricity ratings revealed impaired metaphor recognition and a higher metaphoricity rating for ABS endings compared to controls. More specifically, the healthy controls indicated correct responses (i.e. conjunction ratings: MET yes/yes, NEU yes/no, ABS no/no) on all conditions more frequently than schizophrenia subjects, with the biggest difference in MET comprehension level. Considering the specific error rates, we found that the most common quantitative error that occurred in both groups was related to the figurative misinterpretation of literal meaning (NEU yes/yes), but significantly higher in the clinical group. The second error found, specific for schizophrenia, was related to the problems with figurative meaning understanding with proper recognition of metaphoricity (MET no/yes). At last, the strongest effects specifically manifested in the clinical group were related to the general problems with language comprehension (MET no/no; NEU no/no). Finally, the reaction times were significantly higher in the clinical group during all comparisons.

**Table 2:** Between-group comparisons of the behavioral ratings of comprehensibility and metaphoricity during punchline-based metaphor comprehension task performance

Ratings/type of punchline	Responses (yes/no)		Schizophrenia outpatients (n = 30)			Healthy controls (n = 30)			Between-group differences (Mann–Whitney *U*)		
	Comprehensible?	Metaphoric?	Mean	SD	SE	Mean	SD	SE	W	ES	p
*Comprehensibility*											
MET	Yes	–	54.3	6.46	1.18	58.8	1.52	0.28	201	− 0.553	< 0.001
NEU	Yes	–	57.2	3.58	0.65	59.5	1.01	0.18	215	− 0.521	< 0.001
ABS	No	–	53.7	10.09	1.84	58.3	2.89	0.53	293	− 0.348	0.017
*Metaphoricity*											
MET	–	Yes	53.3	7.81	1.43	57.4	4.12	0.75	269	− 0.401	0.007
NEU	–	No	46.9	17.65	3.22	53.1	13.88	2.54	309	− 0.313	0.035
ABS	–	No	53.7	9.91	1.81	57.8	3.93	0.72	320	− 0.288	0.05
*Correct responses*											
MET	Yes	Yes	50.7	8.52	1.56	56.6	4.22	0.77	210	− 0.533	< 0.001
NEU	Yes	No	44.5	19.13	3.49	52.6	14.1	2.57	255	− 0.432	0.004
ABS	No	No	51.6	10.49	1.92	56.7	4.69	0.86	292	− 0.35	0.019
*Erroneous responses*											
MET	Yes	No	3.6	4.86	0.89	2.2	3.41	0.62	533	0.184	0.206
ABS	Yes	No	2.1	4.12	0.75	1.2	2.17	0.4	513	0.141	0.31
ABS	Yes	Yes	4.2	9.7	1.77	0.5	1.04	0.19	553	0.229	0.07
NEU	Yes	Yes	12.7	17.6	3.21	6.9	13.78	2.52	587	0.304	0.04
MET	No	Yes	2.6	4.11	0.75	0.8	1.09	0.2	593	0.318	0.027
MET	No	No	3.1	5.71	1.04	0.4	1.04	0.19	620	0.378	0.004
NEU	No	No	2.33	2.78	0.51	0.5	0.9	0.16	683	0.518	< 0.001
Reaction times											
*Comprehensibility*											
MET	–	–	2.9	1.94	0.35	1.6	0.92	0.17	668	0.484	0.001
NEU	**–**	–	3	1.9	0.35	1.6	0.69	0.13	667	0.482	0.001
ABS	**–**	–	3	2.09	0.38	1.8	0.81	0.15	638	0.418	0.005
*Metaphoricity*											
MET	–	–	1.4	0.82	0.15	1	0.47	0.09	605	0.346	0.022
NEU	–	–	1.7	0.88	0.16	1.1	0.5	0.09	630	0.4	0.007
ABS	–	–	1.6	0.87	0.16	1.2	0.61	0.11	565	0.257	0.089

Scores of ratings and reaction times were presented as mean ± standard deviation (SD) and standard error (SE) for all types of punchlines (*MET* Metaphoric; *NEU* Neutral; *ABS* Absurd). For ratings sum of responses was presented indicating the story was rated as comprehensible in case of NEU and FUN punchlines, or non-comprehensible in case of ABS punchlines, with max score = 60 (min = 0). Reaction times for ratings are presented in seconds. *ES* Effect size (rank-biserial correlation)

**Fig. 1:**
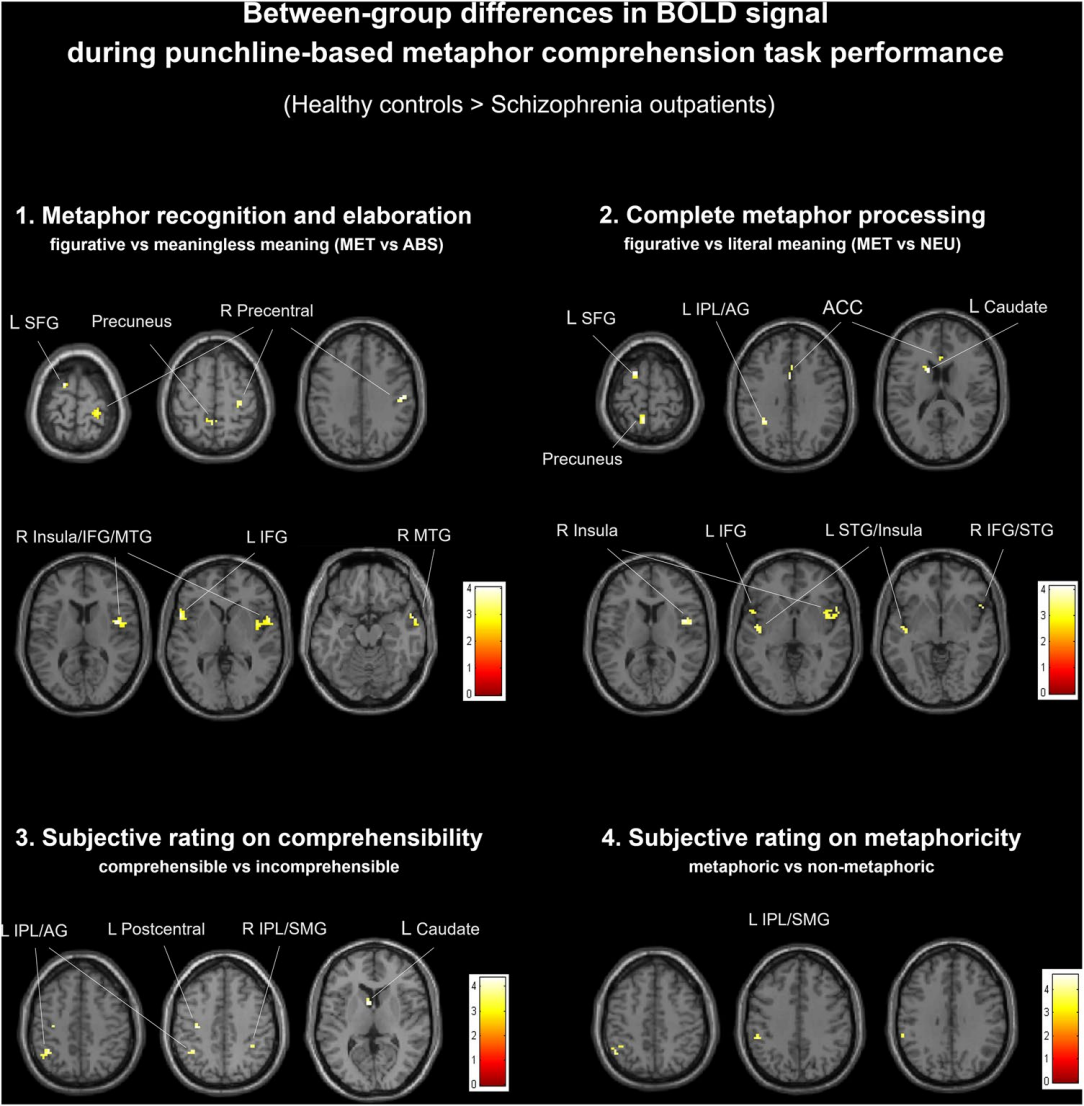
Between-group differences in the brain activations during punchline-based metaphor comprehension task performance**.** Localization of brain regions revealed by between-group contrasts (healthy controls > schizophrenia outpatients) during punchline-based metaphor comprehension task performance. Statistical analysis utilized a non-parametric whole-brain voxel-wise Pseudo-t-test. Significant clusters are thresholded at k ≥ 10 voxels uncorrected at alpha = 0.0010. The visualizations were obtained by XjView toolbox, version 9.6 (http://www.alivelearn.net/xjview)**.** MNI coordination for the presented slices in transversal plane: panel (1) 71, 56, 35, 11, 2, − 16; panel (2) 68, 32, 17, 11, 0, − 4; panel (3) 47, 44, 8; panel (4) 47, 38, 32. *MET* metaphoric, *NEU* neutral, *ABS* absurd, *L* left, *R* right, *ACC* anterior cingulate cortex, *IFG* inferior frontal gyrus, *SFG* superior frontal gyrus, *MTG* middle temporal gyrus, *STG* superior temporal gyrus, *IPL* inferior parietal lobule, *AG* angular gyrus, *SMG* supramarginal gyrus

Between-group differences in brain activations during fMRI punchline-based metaphor comprehension task revealed no significant differences in BOLD signal during incongruity detection and absurd processing (ABS vs NEU) except increased activation in the small cluster (k = 6, Pseudo-t = 3.89) covering the right IFG pars triangularis (MNI x, y, z = 33, 23, 11) in the healthy controls. During metaphor recognition and elaboration (MET vs ABS), schizophrenia outpatients showed decreased activation in the left IFG pars opercularis, left superior frontal gyrus (SFG), interhemispheric, but leftward-shifted, precuneus, as well as in the right insula, right frontal–temporal space, right temporal pole (TP) and middle temporal gyrus (MTG), and right precentral/postcentral gyri. During the complete metaphor processing (MET vs NEU), schizophrenia outpatients showed decreased activation in the left caudate, interhemispheric dorsal ACC, left frontal lobe (IFG pars opercularis, medial frontal gyrus (MFG), SFG), left insula, Heschl and superior temporal gyrus (STG), precuneus, IPL (angular and supramarginal gyri), and in the right insula and right fronto-temporal cluster (IFG/STG). Moreover, the additional analysis of neural correlates of subjective responses on comprehensibility (comprehensible > incomprehensible) and metaphoricity (metaphorical > non-metaphoric) ratings revealed between-group differences (healthy controls > schizophrenia subjects) in several brain regions. Namely, we found the stronger neural response in the left hemisphere related to comprehensible (left caudate, left precentral/postcentral gyri, and bilateral IPL) and metaphoric (L IPL) stimuli in normotypic brains. Importantly, we found no regions with increased activation in the schizophrenia group compared to healthy controls in any of the examined contrasts. For detailed between-group results see Fig. 1 and Table 3.

**Table 3:** Between-group differences in BOLD signal during punchline-based metaphor comprehension task performance

Contrast/brain region	BA	k	MNI			Pseudo-t
			x	y	z	
*Metaphor recognition and elaboration—figurative vs meaningless meaning (MET vs ABS)* Healthy controls > Schizophrenia outpatients						
L Inferior Frontal Gyrus pars opercularis	45	25	− 54	11	2	4.57
L Superior Frontal Gyrus	6	20	− 15	5	71	5.13
Interhemispheric (L > R) Precuneus	7	106	0	− 46	56	4.20
R Insula/Precentral Gyri	13	40	45	2	11	4.32
R Frontal–Temporal space (Insula / Inferior Frontal Gyrus/Temporal Pole / Superior Temporal Gyrus)	22/45	12	57	8	5	4.34
R Middle Temporal Gyrus / Temporal Pole	22	11	57	2	− 16	4.38
R Precentral / Postcentral Gyri	4	45	27	− 28	71	4.24
		31	54	− 19	35	4.03
		24	45	− 16	59	4.00
*Complete metaphor processing—figurative vs literal meaning (MET vs NEU)* Healthy controls > Schizophrenia outpatients						
L Caudate	–	21	− 15	11	17	4.34
Interhemispheric (L > R) dorsal Anterior Cingulate Cortex	24/33	11	0	17	23	3.90
L Inferior Frontal Gyrus pars opercularis	45	11	− 54	11	2	4.25
L Medial Frontal Gyrus / Superior Frontal Gyrus	6	10	− 15	8	68	4.10
L Superior Temporal Gyrus / Heschl Gyrus / Insula	22	22	− 48	− 13	5	3.79
L Precuneus	7	43	− 6	− 52	68	4.13
L Inferior Parietal Lobule (Angular Gyrus)	40	20	− 30	− 55	41	4.04
		14	− 33	− 46	32	3.83
R Insula / Precentral Gyri	13	15	42	2	2	3.89
		24	45	2	11	3.78
R Inferior Frontal Gyrus / Superior Temporal Gyrus	45	11	54	14	− 4	3.82
*Subjective rating on comprehensibility—comprehensible vs incomprehensible* Healthy controls > Schizophrenia outpatients						
L Caudate	–	13	− 6	8	8	4.36
L Precentral / Postcentral Gyri	4	31	− 33	− 19	44	4.17
L Inferior Parietal Lobule (Angular Gyrus)	40	23	− 39	− 49	47	4.05
R Inferior Parietal Lobule (Supramarginal Gyrus)	40	23	36	− 43	44	3.69
*Subjective rating on metaphoricity—metaphoric vs non-metaphoric* Healthy controls > Schizophrenia outpatients						
L Inferior Parietal Lobule (Supramarginal Gyrus)	40	32	− 51	− 37	38	3.97
		26	− 48	− 58	47	3.97
		12	− 60	− 40	32	3.63

List of brain regions revealed by between-group contrasts during punchline-based metaphor comprehension task performance. Statistical analysis utilized a non-parametric whole-brain voxel-wise Pseudo-t-test. Localizations are reported as local maximum threshold with k ≥ 10 voxels extent threshold uncorrected at alpha = 0.0010. *L* left hemisphere, *R* right hemisphere, *BA* Brodmann's area, *k* number of voxels in analyzed cluster size, *MNI* Montreal Neurological Institute coordinates

**Fig. 2:**
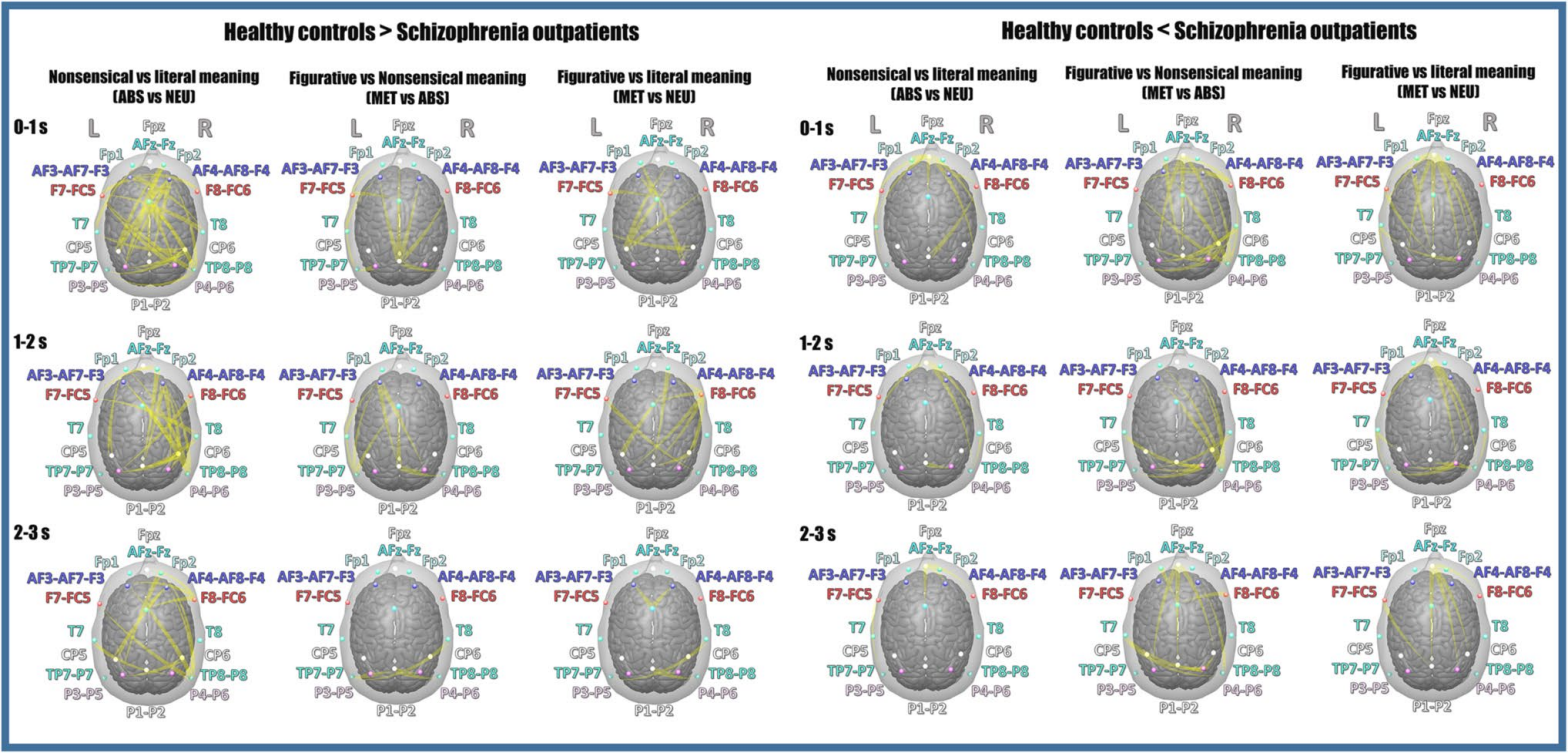
Effective connectivity maps for between-group differences during punchline-based metaphor comprehension task performance. Conjunction maps of effective connectivity during three contrasts: incongruity detection (ABS vs NEU); metaphor recognition and elaboration (MET vs ABS); and complete metaphor processing (MET vs NEU). Information flow in the metaphor-related neural circuit revealed by between-group contrasts presented in 3 epochs (0–1, 1–2, 2–3 s). Arrows reflect the group differences in information flow (source → receiver). *L* left hemisphere, *R* right hemisphere, *Fpz* orbitofrontal cortex (oPFC), *Fp1/Fp2* ventromedial prefrontal cortex (vmPFC), *AFz-Fz* dorsomedial prefrontal cortex (dmPFC), AF3-AF7-F3/AF4-AF8-F4 dorsolateral prefrontal cortex (dlPFC), *F7-FC5/F8-FC6* inferior frontal gyrus (IFG), *T7/T8* anterior temporal lobe (aTL), *TP7-P7/TP8-P8* posterior temporal lobe (pTL), *CP5/CP6* temporoparietal junction (TPJ), *P3-P5/P4-P6* inferior parietal lobule (IPL), *P1-P2* precuneus (Prec)

The EEG–DTF results revealed abundant differences in information flow during punchline-based metaphor comprehension task performance. The investigated metaphor comprehension network was performed with intense information flow and interchangeable directional signal propagation within the bilateral fronto-temporo-parietal (vmPFC/oPFC/dmPFC/IFG/dlPFC–aTL/pTL–IPL/TPJ/Prec) brain regions. The essential differences were related to the diverse discrimination pattern of the three assessed conditions (NEU, ABS, MET) manifested by the opposite directionality of information flow and alternate lateralization of source → receiver activity patterns. The maps of neural propagation are presented for three contrasts: ABS vs NEU, MET vs ABS, and MET vs NEU (Fig. 2). The complete lists containing detailed DTF statistics of significant between-group interaction effects with directions of electrode pairs for each contrast during a 3 s period are presented in Online Resources 2.

## Discussion

In the present study, we investigated functional disturbances and effective connectivity abnormalities related to impaired conventional metaphor processing in schizophrenia outpatients, as obtained with the punchline-based metaphor comprehension task. In line with previous studies (Adamczyk et al. 2016; Pawełczyk et al. 2018; Rapp et al. 2018), we revealed that the clinical group was less accurate in conventional metaphor recognition and elaboration, as compared to controls. We found that the difficulties with comprehension of figurative meaning were related either to the (i) general problems with language comprehension, (ii) problems with understanding of figurative meaning within context, but with proper recognition of conventional metaphors, or iii) with more often figurative misinterpretation of literal meaning. Regarding neural substrates of the observed impairment, the fMRI data revealed that the diminished ability to comprehend conventional metaphors observed in schizophrenia is related to the bilateral, but dominantly leftward-shifted fronto-temporo-parietal brain regions hypofunction (i.e. ACC, IFG, SFG, STG, IPL and precuneus) accompanied with suppressed activity in the left caudate. More specifically, the analysis concerning the subjective ratings on the comprehensibility and metaphoricity consistently revealed the dominant left hemisphere hypofunction, especially related to the suppressed left IPL activity in clinical subjects. However, diminished activity in the right fronto-temporal space (insula, IFG, TP, MTG/STG), precuneus, or IPL observed during conventional metaphor processing in schizophrenia also should be noted as an essential part of the bilateral figurative language network abnormalities in the schizophrenic brain. Complementary, EEG results revealed alternate paths of cortical communication and reversed lateralization of sources and receivers which may be regarded as a crucial neural substrate of abnormal metaphor processing in schizophrenia and most probably, together with observed fMRI hypofunction serve as a neural ground of behavioral manifestation of the impairment.

### On the Role of Left and Right Hemisphere Hypofunction—fMRI Data

In the clinical group, we observed the bilateral, but dominantly LH hypofunction, observed in the caudate, cingulate cortex (interhemispheric ACC), and in frontal (IFG, SFG) and parietal (IPL, interhemispheric precuneus) lobes. Importantly, it should be pointed out however, this was accompanied by diminished activation of the right insula, fronto-temporal lobe (IFG, TP, MTG/STG), and precentral/postcentral gyri. Although, the essential role of the (dominant) suppressed LH activity as the neural substrate of abnormal conventional metaphor processing in schizophrenia was confirmed by analysis of subjective linguistic content comprehensibility and metaphoricity ratings. Indeed, specific hypoactivation was observed in the left caudate, precentral/postcentral gyri, and bilateral IPL concerning the comprehensibility. Consecutively, the subjective values on metaphoric content recognition in the clinical group were related to the suppressed activity in the left IPL, in more detail, the left supramarginal gyrus.

First of all, the frontal hypofunction of the interhemispheric dorsal ACC and left MFG/SFG is consistent with previous data on diminished humor comprehension as another impaired figurative aspect of language in schizophrenia (Adamczyk et al. 2017; Berger et al. 2018; Vrticka et al. 2013). This could reflect the cognitive deficiencies, e.g. suppression of executive control, error detection and conflict monitoring system (Gauvin et al. 2016; Swick and Turken 2002), the processes seemingly affected in schizophrenia and influencing task performance (e.g. longer reaction times). Consistently, the presented findings on hypofunction of the left caudate, especially results on the comprehensibility ratings indicate the important role of the disturbances in the basal ganglia language cognitive control system (Gil Robles et al. 2005; Wang et al. 2013) in schizophrenia. This is in line with previous studies on the left caudate engagement in conventional metaphor processing (Rapp et al. 2012), abnormal (weaker) hemispheric specialization of the left caudate and its disrupted inter-hemispheric connectivity with cortical regions (Mueller et al. 2015), aberrant caudate-PFC connectivity related to altered humor processing (Berger et al. 2018), and that on the relationship between caudate hypofunction and more severe psychopathological symptoms and cognitive deficits (Bernard et al. 2017) in schizophrenia.

Second, our finding on the left IFG hypofunction adds to the previous research on the crucial role of this region in the processing of conventional metaphors (Rapp et al. 2012). However, it should be noted, that the diminished activity was observed also in the right IFG (i.e. as part of the fronto-temporal space cluster). Importantly, the left IFG is recruited in both conventional and novel metaphors processing (Reyes-Aguilar et al. 2018) and is regarded as a key region for the integration of single words into a meaningful sentence (Badre and Wagner 2007; Menenti et al. 2009) and non-literal meanings into a context (Bambini et al. 2016; Rapp et al. 2007). The right IFG is considered a key region for disentangling non-literal meanings (Kircher et al. 2007; Rapp et al. 2004), set-shifting and generating possible relations for abstract concepts (Bohrn et al. 2012).

Third, we found a diminished activity of the left IPL in schizophrenia observed either during conventional metaphor processing (MET vs NEU), its subjective comprehensibility, and especially, recognition of metaphoricity ratings. At the same time, the hypoactivation in the right IPL was solely related to the subjective language content comprehensibility, along with the LH hypoactivity (caudate, precentral/postcentral gyri, IPL) in schizophrenia. Previous studies revealed the right IPL activation within the fronto-parietal attentional network during literal language processing and its enhanced activation during semantic combination of distant concepts (Bohrn et al. 2012), processes seemingly engaged in the comprehensibility of linguistic content. On the other hand, the left IPL was recognized to play an important role in the processing of figurative expressions (Rapp and Wild 2011; Yang et al. 2009), i.e. it integrates individual semantic concepts within a given context (Binder and Desai 2011; Obert et al. 2014). Thus, we can suggest that the diminished activity of the bilateral, but dominant left fronto-temporo-parietal regions (IFG, insula, TP, STG, Heschl Gyrus and IPL) may be associated with the disturbances of contextual cues processing, believed to be essential for the observed figurative language impairment in schizophrenia (Bambini et al. 2020; Kuperberg 2010; Kuperberg et al. 1998).

Fourth, we found diminished activity within the interhemispheric, but dominantly leftward-shifted cluster covering the precuneus in the clinical group. The abnormal activity of this region during metaphor processing was previously detected in schizophrenia, but results are inconsistent (Kircher et al. 2007; Mashal et al. 2014). However, presented findings indicate that precuneus may be considered as an important region for deficiencies in figurative language comprehension. The disruptions of metaphor understanding could be associated with lesser familiarity and/or more effort during (even salient and conventional) figurative meaning processing in schizophrenia (Schmidt and Seger 2009). Conversely, enhanced activity of precuneus in healthy controls may reflect their better abilities of theory of mind and first/third-person perspective taking (Parola et al. 2020; Yuan et al. 2018), more effective attention shifting related to self-referential thoughts, judgments and memory retrieval (Binder et al. 2009; Binder and Desai 2011; Bohrn et al. 2012), better literal language comprehension (Stringaris et al. 2007, 2006) and metaphor understanding (Schmidt and Seger 2009), and better ability to use (metaphor-related) mental imagery (Obert et al. 2014; Yang et al. 2009).

At last, apart from the dominant LH hypoactivity, the presented results revealed also suppression of activity in several RH brain regions previously recognized as a part of bilateral figurative language network in the general population (Bohrn et al. 2012; Rapp et al. 2012; Reyes-Aguilar et al. 2018). However, since conventional metaphor may be regarded as the salient meaning of language, we found unexpectedly (Bohrn e al. 2012, Rapp et al. 2012) that the differences between healthy controls and schizophrenia subjects were related to the suppressed activity in the right fronto-temporal space (insular cortex, IFG, TP, MTG/STG), precentral/postcentral gyri and IPL during metaphor processing and comprehension. Thus, even if only conventional (but not novel) metaphors were investigated in the present study, the bilateral brain activity abnormalities in schizophrenia should be emphasized as a neural substrate of the observed impairment. This discrepancy with literature data indicating expected solely LH activity during conventional (salient) metaphors and idioms processing (Bohrn et al. 2012; Rapp et al., 2012; Reyes-Aguilar et al. 2018) may be assumed as an effect of the design of the punchline-based task, and/or more specifically to Polish language characteristics, but further research on both conventional and novel metaphors processing in schizophrenia is required to resolve this question. Yet, in line with the literature data, the hypoactivity of the insula, fronto-temporal, and precentral/postcentral gyri in RH may be related to the less efficient higher-order cognitive and semantic processes in schizophrenia, e.g. less efficient searching for a semantic relationship between alternative (literal vs figurative) meanings of metaphor (Butti and Hof 2010; Hagoort 2019; Rapp et al. 2012; Romero Lauro et al. 2013; Yang and Shu 2016).

Hence, regarding the presented findings on diminished brain activity in schizophrenia, it should be pointed out, that in the clinical studies on schizophrenia, one of the most evident and replicable fMRI findings is hypofunction of the executive network related to the cognitive impairment or vice versa, higher brain activity related to lesser impairment (Smucny et al. 2019; Wolf et al. 2015). Therefore, we may conclude, that the presented hypofunction of the bilateral fronto-parietal regions may be related to the observed impairment at the behavioral level, which is supported by diminished source activity revealed by EEG-DTF data, and in line with the previous study on humor (Adamczyk et al. 2017; Berger et al. 2018) and on impaired word processing in schizophrenia (Ragland et al. 2004).

### Metaphor Comprehension and the Reversed Directionality of Information Flow in Schizophrenia: EEG Data

Presented EEG results complement our fMRI findings, as we observed enhanced source activity of the IFG, IPL/TPJ and precuneus during metaphors processing in healthy controls, but not in schizophrenia. The absence of increased source activity may be regarded as the primal cause of the observed hypofunction in these regions observed in the fMRI experiment. Noteworthy, a specific difference in the connectome was observed between the groups. Namely, most of the connections that in healthy subjects showed higher activity for the absurd (ABS), but not literal (NEU) meaning processing were characterized by reversed connectivity values in schizophrenia outpatients, i.e. higher flow for processing of literal (NEU) meaning, but not activated by meaningless sentences (ABS). The same opposite pattern was observed during figurative meaning processing (MET). The connections activated by metaphors in healthy controls were activated by meaningless sentences in schizophrenia outpatients, but not by metaphoric content.

Moreover, along with the weakened information flow in the LH during metaphor processing, we found an opposite pattern reflected by a rightward shift of sources in the clinical group. This was manifested by enhanced activity of the RIFG, RIPL/TPJ, RPTL together with excessive and long-lasting orbitofrontal (oPFC) source engagement in schizophrenia. Our effective connectivity results clearly demonstrate that the dominant localization of sources and thus the direction of information were vividly reversed and strongly supports the abnormal language lateralization in schizophrenia (Chakrabarty et al. 2014; Leroux et al. 2017; Mitchell and Crow 2005).

Noteworthy, EEG effective connectivity results were visibly more precise and sensitive to reveal both specific bi-directional between-group differences than fMRI that showed only hypoactivated, but no hyperactivated regions in clinical subjects (i.e. schizophrenia > controls). This apparent difference could be attributed to unique characteristics of both methods (Abreu et al. 2018). It should be noted, that they pick up different aspects of neural activity and we do not expect similar results in this regard, but rather more deep investigation into focal activations (fMRI) and neural communication (DTF). Therefore, the results from both methods should be interpreted in complementary fashion.

Yet, in line with GSH predictions on salient vs non-salient content processing, our results on incongruity detection (ABS vs NEU) indicate a bilateral source activity, with some apparent rightward dominance in healthy controls, which differs from that observed in schizophrenia. The spatiotemporal information flow pattern during absurd processing in the normotypical brain is characterized by rapid and long-lasting (0–3 s) outflows from left dmPFC and TPJ together with the dominant activity of RH fronto-temporo-parietal sources (RvmPFC, RIFG, RpTL, RTPJ) towards LH fronto-parietal receivers (IFG, dlPFC, IPL, precuneus). Conversely, in schizophrenia, absurd processing recruits an alternative neural circuit, i.e. dominant oPFC, left IFG, or precuneus sources. These observations indicate alternate interhemispheric information exchange in schizophrenia and replicate our previous findings from humor on impaired incongruity detection process (Adamczyk et al. 2019, 2017) and these on abnormal language discrimination task processing (Iakimova et al. 2005; Nestor et al. 1997; Salisbury et al. 2002; Schneider et al. 2015). Thus, more active RH sources in healthy controls may be regarded as associated with higher efficacy of non-salient content (ABS) processing and incongruity detection process (e.g. no clear associations between distant meanings) and/or more excessive language processing (e.g. an intense search for the possibility of finding new figurative meaning within given non-salient nonsensical sentences).

Furthermore, in agreement with the GSH theory on the lateralization switch between salient and non-salient content processing, our connectivity result shows it visible when compared incongruity detection (ABS vs NEU) with those of metaphor recognition and elaboration (MET vs ABS; MET vs NEU). In the clinical group, the result of conventional metaphor processing showed consistently reversed lateralization. In schizophrenia, the information flow during metaphor comprehension is more excessive, engaging alternate circuits but with evidently reversed lateralization, similar to absurd processing in healthy controls. Metaphor recognition and elaboration (MET vs ABS) in schizophrenia was associated with excessive and long-lasting (0–3 s) activation of RH sources in the fronto-temporo-parietal regions (RIFG, Ra/pTL, RTPJ, RIPL), with a specifically enhanced connectivity of the oPFC to the precuneus and bilateral IPL/TPJ. In healthy controls, the most pronounced and long-lasting source in metaphor recognition and processing was the precuneus (0–3 s) together with essential LH source activation of the fronto-temporal regions, i.e. IFG, dlPFC, aTL, pTL, IPL. Consistently, results on complete metaphor processing (MET vs NEU) revealed that the most essential difference between schizophrenia and healthy controls is the schizophrenia-characteristic pronounced and long-lasting information flows from the oPFC source to the widespread LH fronto-temporal (LIFG, LATL) and RH parietal receivers (precuneus, pTL, IPL, TPJ), accompanied by the specific engagement of the RIPL source activity. In healthy controls, we observed characteristic engagement of the LIFG and LTPJ activity that was propagated intrahemispherically to the RH frontal receivers at the early stage of processing (0–1 s). Importantly, at 1–2 s of MET processing, toward long-lasting LTPJ activity, the specific RH source activity of RVMPFC, RIFG and RTPJ has been observed, with later (2–3 s) precuneus and DMPFC activity. These findings, complementary to these from fMRI, indicate that conventional metaphors processing (assumed to be a salient and familiar meaning) in the normotypical brain involves bi-hemispheric communication comprised of enhanced LH sources activity at the early stage of processing, but also further selective RH source engagement. In general, these data support the step-by-step GSH model of metaphor processing (Giora 2007; Rapp et al. 2012), i.e. bilateral engagement of brain structures involved in former LH automatic literal processing (LIFG, LTPJ) followed by latter RH figurative semantics (RIFG, RVMPFC, RTPJ), even if related to the commonly known metaphorical meanings.

Finally, regarding precise localization of the brain regions, the presented EEG data should be considered with caution as they represent the sensor-level effective connectivity. Additional source localization analysis in future studies could provide more insight into the abnormalities of brain activity in schizophrenia.

### RH and Figurative Language Processing

In line with recent studies on non-literal language in the normotypical brain (Bohrn et al. 2012; Diaz and Eppes 2018; Obert et al. 2014; Rapp et al. 2012; Reyes-Aguilar et al. 2018), our EEG and fMRI results indicate that both hemispheres were involved in literal and figurative language, and we revealed that conventional metaphors dominantly activate the left-lateralized fronto-temporo-parietal network with exceptional but essential engagement of the RH fronto-parietal regions (RvmPFC, RIFG/insula, RTPJ, RIPL). The presented EEG and fMRI results revealed that along with the previous findings on essential RIFG involvement in the processing of novel/unfamiliar/non-salient content, its activity is also necessary for conventional/familiar metaphor processing. Importantly, some authors suggest that the magnitude of RIFG activity may reflect an increasing level of difficulty in decoding the figurative meaning (Bohrn et al. 2012) and recruitment of the attentional and working memory areas due to cognitive demand for comprehension (Vigneau et al. 2011), what altogether may reflect more effective higher-order language comprehension processes in healthy controls. All above is consistent with our EEG findings, as in healthy controls RIFG source activation is apparently more rapid, enhanced and long-lasting during non-salient/meaningless content processing (ABS vs NEU) than observed specific and phasic (1–2 s) response during salient/conventional metaphors processing (MET vs NEU). Noteworthy, fMRI results revealed the small RIFG hyperactivity clusters in healthy controls during absurd processing (ABS vs NEU; IFG pars triangularis k = 6) and as part of the fronto-temporal cluster during conventional metaphor processing (MET vs ABS; k = 12; MET vs NEU; k = 11).

It should be pointed out that conventional vs novel metaphors were believed to be associated with enhanced LH (LIFG, LTL, LIPL) or RH (RIFG, RACC, RPFC) activity, respectively (Bohrn et al. 2012; Rapp et al. 2012; Reyes-Aguilar et al. 2018). Our result showed dynamic composition of hemispheric communication and bi-lateral engagement of widespread neural networks is required for an effective figurative language comprehension process. Hence, our findings seem to be consistent with the GSH (Giora 1997) as we found excessively dominant RH activation during processing of anomalous semi-figurative expressions contained in the ABS condition in healthy controls accompanied with dominant LH activation during MET condition processing.

### Hemispheric Specificity of the Abnormal Brain Activity and Reversed Information Flow During Humor and Metaphor Processing in Schizophrenia

The presented effective connectivity findings, as a continuation of our previous investigation on humor (Adamczyk et al. 2019), clearly indicate the dissociation and specificity of humor and conventional metaphor processing in normotypic brains and the dissociation of the neural underpinning of these deficits in schizophrenia. Namely, when compared to healthy controls, specificity in humor and conventional metaphor processing is related to dominant hypoactivation of the RH and the LH, respectively. In particular, at the neural level this is related to the evident dissociation of lateralization-shift on effective connectivity during humor (LH < RH) and conventional metaphor (LH > RH) processing in normotypic brain, consistently accompanied with the opposite (reversed) pattern of neural activity observed in schizophrenia, i.e. leftward-shifted (LH > RH) source localizations during humor processing, and the rightward-shifted (LH < RH) pattern during metaphor processing. Thus, GSH prediction on lateralization shift in a healthy population is coherent with our findings which substantially extended recent knowledge on attenuated brain activity and reversed lateralization of information flow in humor (non-salient content) and conventional metaphors (salient content) in schizophrenia. In conclusion, we indicate that differential engagement of neural circuits and altered cortical information flow may serve as an essential neural substrate of figurative speech impairment in schizophrenia, which is manifested as a compromised incongruity detection, incongruity resolution and elaboration process.

Finally, the presented results support the evidence on the reversed lateralization of the language neural network (Leroux et al. 2015, 2017; Sheng et al. 2013; Son et al. 2017) in schizophrenia. Moreover, we indicate that differential engagement of neural circuits (i.e. reversed effective connectivity) and altered cortical information flow may serve as a neural substrate of figurative speech impairment in schizophrenia, which is manifested as a compromised incongruity detection, metaphor recognition, and elaboration process. All this may be due to the abnormal neurodevelopment in schizophrenia, e.g. altered synaptic pruning, suppressed myelinization, which primal processes provide to failures of functional integration of neural circuits (Kelly et al. 2018) and in general, to the brain disconnection in schizophrenia (Friston et al. 2016) manifested by the existence of compensatory recruitment of alternative neural circuits (Cobia et al. 2012; Mashal et al. 2014; Tan et al. 2006) and characteristic reversed language lateralization (Chakrabarty et al. 2014; Leroux et al. 2017; Mitchell and Crow 2005).

### Conclusions

The present study extends the knowledge on the neural substrates of impaired metaphor comprehension in schizophrenia. Our results revealed that behavioral manifestations of difficulties in comprehension of conventional metaphors in clinical subjects were accompanied—on a neural level—by hypoactivation of the bilateral, but leftward-shifted brain regions and recruitment of an alternative neural circuit and alternate interhemispheric information exchange. We found reversed connectivity patterns in fronto-temporo-parietal regions, i.e. altered RH source activity during incongruity detection and processing of absurd meanings, and diminished source activity of LH with notably reversed lateralization of the information flow during metaphor processing. Moreover, we found a dissociation between the lateralization shift of neural substrates of humor and metaphor impairment in schizophrenia, both related to the reversed information flows pattern, as compared to controls.

## Supplementary Information

Below is the link to the electronic supplementary material.

Supplementary file1 (DOCX 14 kb)

Supplementary file2 (DOCX 39 kb)

## Data Availability

Data and material (anonymized) available on request of Authors.

## Code Availability

Not applicable.

**Author Contributions [CRediT].**

PA: Conceptualization, Investigation, Methodology, Software, Visualization, Writing—Original draft preparation, Writing—Review & Editing, Supervision, Project administration, Funding acquisition; MJ: Investigation, Formal analysis, Visualization, Writing—Review & Editing; OP: Investigation, Methodology; TSL: Methodology, Software, Formal analysis, Writing—Review & Editing; PB: Investigation, Methodology; MW: Conceptualization, Methodology, Data Curation, Software, Formal analysis, Supervision, Writing—Review & Editing.
