# Supplementary material for: On the Role of Bilateral Brain Hypofunction and Abnormal Lateralization of Cortical Information Flow as Neural Underpinnings of Conventional Metaphor Processing Impairment in Schizophrenia: An fMRI and EEG Study
Source: Brain Topogr. 2021 May 10;34(4):537–54. doi: 10.1007/s10548-021-00849-x (PMC8195899; doi:10.1007/s10548-021-00849-x)
Supplement: Supplementary file 1 — Supplementary file1 (DOCX 14 kb) [file 10548_2021_849_MOESM1_ESM.docx]

**On the role of left hemisphere hypofunction and abnormal lateralization of cortical information flow as neural underpinnings of conventional metaphor processing impairment in schizophrenia - an fMRI and EEG study**

***Brain Topography***

Przemysław Adamczyk1✉, Martin Jáni1,2, Olga Płonka1, Tomasz S Ligeza1, Piotr Błądziński3, Miroslaw Wyczesany1

[1] Institute of Psychology, Jagiellonian University, Krakow, Poland; [2] Department of Psychiatry, Faculty of Medicine, Masaryk University and University Hospital Brno, Brno, Czech Republic; [3] Department of Community Psychiatry, Chair of Psychiatry, Medical College, Jagiellonian University, Krakow, Poland; [*] The study was conducted by the Krakow Schizophrenia Research Group, Krakow, Poland; [✉] Corresponding author: Dr Przemysław Adamczyk; Institute of Psychology, Jagiellonian University, Krakow, Poland; email: przemyslaw.adamczyk@uj.edu.pl ; additional email: [przemyslaw.adamczyk@mailplus.pl](mailto:przemyslaw.adamczyk@mailplus.pl)

**Online Resource 1 Ratings of metaphoricity and comprehensibility for chosen stories in pre-selective judgements.**

| **Type of punchline** | **Metaphoricity rating** | | **Comprehensibility rating** | |
| --- | --- | --- | --- | --- |
|  | Mean | ±SD | Mean | ±SD |
| Metaphorical (MET) | 8.36 | 0.29 | 8.40 | 0.45 |
| Neutral (NEU) | 1.94 | 0.56 | 8.51 | 0.36 |
| Abstract (ABS) | 1.62 | 0.46 | 1.56 | 0.50 |

Scores of pre-selective ratings assessed by a panel of judges were presented as mean ± standard deviation (SD) of metaphoricity and comprehensibility ratings for all types of punchlines (n=30).
