# Supplementary material for: On the Role of Bilateral Brain Hypofunction and Abnormal Lateralization of Cortical Information Flow as Neural Underpinnings of Conventional Metaphor Processing Impairment in Schizophrenia: An fMRI and EEG Study
Source: Brain Topogr. 2021 May 10;34(4):537–54. doi: 10.1007/s10548-021-00849-x (PMC8195899; doi:10.1007/s10548-021-00849-x)
Supplement: Supplementary file 2 — Supplementary file2 (DOCX 39 kb) [file 10548_2021_849_MOESM2_ESM.docx]

**On the role of bilateral brain hypofunction and abnormal lateralization of cortical information flow as neural underpinnings of conventional metaphor processing impairment in schizophrenia - an fMRI and EEG study**

***Brain Topography***

Przemysław Adamczyk1✉, Martin Jáni1,2, Tomasz S Ligeza1, Olga Płonka1, Piotr Błądziński3, Miroslaw Wyczesany1

[1] Institute of Psychology, Jagiellonian University, Krakow, Poland; [2] Department of Psychiatry, Faculty of Medicine, Masaryk University and University Hospital Brno, Brno, Czech Republic; [3] Community Psychiatry and Psychosis Research Center, Chair of Psychiatry, Medical College, Jagiellonian University, Krakow, Poland; [*] The study was conducted by the Krakow Schizophrenia Research Group, Krakow, Poland; [✉] Corresponding author: Dr Przemysław Adamczyk; Institute of Psychology, Jagiellonian University, Krakow, Poland; email: przemyslaw.adamczyk@uj.edu.pl ; additional email: [przemyslaw.adamczyk@mailplus.pl](mailto:przemyslaw.adamczyk@mailplus.pl)

**Online Resource 2 DTF statistics for between-group interaction effects**

| **A. DTF statistics for incongruity detection contrast – ABS vs NEU** | | | | | | | | | |
| --- | --- | --- | --- | --- | --- | --- | --- | --- | --- |
| **Direction of information flow (source_to_receiver)** | | | | **Between-group Interaction effects** | | | | | |
| **Time window** | **Electrodes** | **ROI to ROI** | | **Standardized Beta Coefficients** | | **Confidence Intervals** | | **Marginal R2** | |
| **0 – 1 s** |  | **Healthy controls > Schizophrenia outpatients** | | | | | | | |
|  | AFz-Fz→P3-P5 | dmPFC_to_LIPL | | 0,25 | | 0.12 - 0.37 | | 0,033 | |
|  | AFz-Fz→P1-P2 | dmPFC_to_Prec | | 0,26 | | 0.16 - 0.36 | | 0,034 | |
|  | AFz-Fz→AF4-AF8-F4 | dmPFC_to_RdlPFC | | 0,21 | | 0.1 - 0.31 | | 0,028 | |
|  | AFz-Fz→P4-P6 | dmPFC_to_RIPL | | 0,22 | | 0.09 - 0.34 | | 0,029 | |
|  | AFz-Fz→CP6 | dmPFC_to_RTPJ | | 0,36 | | 0.18 - 0.53 | | 0,041 | |
|  | AFz-Fz→Fp2 | dmPFC_to_RvmPFC | | 0,31 | | 0.13 - 0.5 | | 0,036 | |
|  | T7→Fpz | LaTL_to_oPFC | | 0,46 | | 0.24 - 0.67 | | NA | |
|  | AF3-AF7-F3→T8 | LdlPFC_to_RaTL | | 0,25 | | 0.12 - 0.38 | | 0,032 | |
|  | F7-FC5→AF4-AF8-F4 | LIFG_to_RdlPFC | | 0,17 | | 0.07 - 0.27 | | 0,021 | |
|  | CP5→AF4-AF8-F4 | LTPJ_to_RdlPFC | | 0,23 | | 0.09 - 0.37 | | 0,065 | |
|  | CP5→Fp2 | LTPJ_to_RvmPFC | | 0,39 | | 0.15 - 0.62 | | NA | |
|  | T8→F7-FC5 | RaTL_to_LIFG | | 0,28 | | 0.12 - 0.44 | | 0,073 | |
|  | AF4-AF8-F4→P3-P5 | RdlPFC_to_LIPL | | 0,18 | | 0.09 - 0.28 | | 0,014 | |
|  | AF4-AF8-F4→P1-P2 | RdlPFC_to_Prec | | 0,14 | | 0.06 - 0.21 | | 0,008 | |
|  | F8-FC6→AF3-AF7-F3 | RIFG_to_LdlPFC | | 0,3 | | 0.2 - 0.4 | | 0,032 | |
|  | P4-P6→F7-FC5 | RIPL_to_LIFG | | 0,25 | | 0.13 - 0.38 | | 0,023 | |
|  | TP8-P8→AFz-Fz | RpTL_to_dmPFC | | 0,33 | | 0.21 - 0.45 | | 0,06 | |
|  | TP8-P8→AF3-AF7-F3 | RpTL_to_LdlPFC | | 0,29 | | 0.19 - 0.39 | | 0,068 | |
|  | TP8-P8→F7-FC5 | RpTL_to_LIFG | | 0,22 | | 0.1 - 0.35 | | 0,058 | |
|  | TP8-P8→TP7-P7 | RpTL_to_LpTL | | 0,21 | | 0.08 - 0.34 | | 0,031 | |
|  | TP8-P8→AF4-AF8-F4 | RpTL_to_RdlPFC | | 0,29 | | 0.19 - 0.39 | | 0,04 | |
|  | CP6→T7 | RTPJ_to_LaTL | | 0,39 | | 0.18 - 0.6 | | NA | |
|  | CP6→P3-P5 | RTPJ_to_LIPL | | 0,31 | | 0.16 - 0.46 | | 0,039 | |
|  | CP6→TP7-P7 | RTPJ_to_LpTL | | 0,26 | | 0.1 - 0.42 | | 0,045 | |
|  | Fp2→P3-P5 | RvmPFC_to_LIPL | | 0,25 | | 0.1 - 0.4 | | 0,054 | |
|  |  | **Healthy controls < Schizophrenia outpatients** | | | | | | | |
|  | F7-FC5→TP7-P7 | LIFG_to_LpTL | | -0,22 | | -0.35 - -0.1 | | 0,017 | |
|  | F7-FC5→Fp1 | LIFG_to_LvmPFC | | -0,29 | | -0.47 - -0.12 | | 0,053 | |
|  | Fp1→Fpz | LvmPFC_to_oPFC | | -0,4 | | -0.6 - -0.19 | | NA | |
|  | Fpz→AFz-Fz | oPFC_to_dmPFC | | -0,38 | | -0.55 - -0.21 | | 0,043 | |
|  | Fpz→T7 | oPFC_to_LaTL | | -0,43 | | -0.66 - -0.21 | | NA | |
|  | Fpz→AF3-AF7-F3 | oPFC_to_LdlPFC | | -0,25 | | -0.38 - -0.12 | | 0,037 | |
|  | Fpz→F7-FC5 | oPFC_to_LIFG | | -0,49 | | -0.65 - -0.32 | | 0,064 | |
|  | Fpz→T8 | oPFC_to_RaTL | | -0,4 | | -0.63 - -0.17 | | NA | |
|  | Fpz→TP8-P8 | oPFC_to_RpTL | | -0,39 | | -0.55 - -0.22 | | 0,084 | |
|  | P1-P2→F8-FC6 | Prec_to_RIFG | | -0,23 | | -0.34 - -0.13 | | 0,028 | |
|  | AF4-AF8-F4→F8-FC6 | RdlPFC_to_RIFG | | -0,16 | | -0.25 - -0.06 | | 0,009 | |
| **1 – 2 s** |  | **Healthy controls > Schizophrenia outpatients** | | | | | | | |
|  | CP5→Fp1 | LTPJ_to_LvmPFC | | 0,4 | | 0.16 - 0.64 | | NA | |
|  | T8→F7-FC5 | RaTL_to_LIFG | | 0,28 | | 0.12 - 0.44 | | 0,073 | |
|  | T8→P3-P5 | RaTL_to_LIPL | | 0,44 | | 0.29 - 0.6 | | 0,13 | |
|  | AF4-AF8-F4→P1-P2 | RdlPFC_to_Prec | | 0,13 | | 0.05 - 0.2 | | 0,008 | |
|  | AF4-AF8-F4→P4-P6 | RdlPFC_to_RIPL | | 0,18 | | 0.08 - 0.27 | | 0,015 | |
|  | F8-FC6→P3-P5 | RIFG_to_LIPL | | 0,22 | | 0.1 - 0.34 | | 0,049 | |
|  | TP8-P8→AFz-Fz | RpTL_to_dmPFC | | 0,42 | | 0.3 - 0.54 | | 0,06 | |
|  | TP8-P8→AF3-AF7-F3 | RpTL_to_LdlPFC | | 0,27 | | 0.17 - 0.37 | | 0,068 | |
|  | TP8-P8→Fp1 | RpTL_to_LvmPFC | | 0,32 | | 0.14 - 0.5 | | 0,062 | |
|  | TP8-P8→AF4-AF8-F4 | RpTL_to_RdlPFC | | 0,3 | | 0.2 - 0.4 | | 0,04 | |
|  | CP6→AFz-Fz | RTPJ_to_dmPFC | | 0,35 | | 0.2 - 0.5 | | 0,084 | |
|  | CP6→AF3-AF7-F3 | RTPJ_to_LdlPFC | | 0,24 | | 0.11 - 0.37 | | 0,057 | |
|  | CP6→P3-P5 | RTPJ_to_LIPL | | 0,28 | | 0.12 - 0.43 | | 0,039 | |
|  | CP6→P1-P2 | RTPJ_to_Prec | | 0,4 | | 0.27 - 0.52 | | 0,07 | |
|  | CP6→AF4-AF8-F4 | RTPJ_to_RdlPFC | | 0,35 | | 0.22 - 0.49 | | 0,033 | |
|  | CP6→P4-P6 | RTPJ_to_RIPL | | 0,29 | | 0.16 - 0.43 | | 0,034 | |
|  | Fp2→F7-FC5 | RvmPFC_to_LIFG | | 0,3 | | 0.14 - 0.46 | | 0,082 | |
|  | Fp2→P1-P2 | RvmPFC_to_Prec | | 0,32 | | 0.2 - 0.44 | | NA | |
|  |  | **Healthy controls < Schizophrenia outpatients** | | | | | | | |
|  | Fpz→T7 | oPFC_to_LaTL | | -0,4 | | -0.63 - -0.16 | | NA | |
|  | Fpz→T8 | oPFC_to_RaTL | | -0,38 | | -0.61 - -0.15 | | NA | |
|  | Fpz→TP8-P8 | oPFC_to_RpTL | | -0,38 | | -0.55 - -0.21 | | 0,084 | |
|  | P1-P2→P4-P6 | Prec_to_RIPL | | -0,19 | | -0.29 - -0.09 | | 0,045 | |
|  | T8→TP8-P8 | RaTL_to_RpTL | | -0,28 | | -0.43 - -0.12 | | 0,038 | |
| **2 – 3 s** |  | **Healthy controls > Schizophrenia outpatients** | | | | | | | |
|  | AFz-Fz→AF4-AF8-F4 | dmPFC_to_RdlPFC | | 0,22 | | 0.11 - 0.33 | | 0,028 | |
|  | T7→P4-P6 | LaTL_to_RIPL | | 0,29 | | 0.14 - 0.45 | | 0,091 | |
|  | CP5→AF4-AF8-F4 | LTPJ_to_RdlPFC | | 0,27 | | 0.13 - 0.41 | | 0,065 | |
|  | F8-FC6→AFz-Fz | RIFG_to_dmPFC | | 0,31 | | 0.19 - 0.43 | | 0,03 | |
|  | F8-FC6→AF3-AF7-F3 | RIFG_to_LdlPFC | | 0,23 | | 0.14 - 0.33 | | 0,032 | |
|  | F8-FC6→Fpz | RIFG_to_oPFC | | 0,28 | | 0.11 - 0.45 | | 0,046 | |
|  | TP8-P8→AFz-Fz | RpTL_to_dmPFC | | 0,27 | | 0.15 - 0.39 | | 0,06 | |
|  | TP8-P8→AF3-AF7-F3 | RpTL_to_LdlPFC | | 0,22 | | 0.12 - 0.32 | | 0,068 | |
|  | TP8-P8→F7-FC5 | RpTL_to_LIFG | | 0,25 | | 0.12 - 0.37 | | 0,058 | |
|  | TP8-P8→AF4-AF8-F4 | RpTL_to_RdlPFC | | 0,36 | | 0.26 - 0.46 | | 0,04 | |
|  | TP8-P8→Fp2 | RpTL_to_RvmPFC | | 0,31 | | 0.13 - 0.49 | | 0,035 | |
|  | CP6→P3-P5 | RTPJ_to_LIPL | | 0,27 | | 0.12 - 0.42 | | 0,039 | |
|  | Fp2→P3-P5 | RvmPFC_to_LIPL | | 0,31 | | 0.17 - 0.46 | | 0,054 | |
|  |  | **Healthy controls < Schizophrenia outpatients** | | | | | | | |
|  | F7-FC5→TP7-P7 | LIFG_to_LpTL | | -0,22 | | -0.34 - -0.1 | | 0,017 | |
|  | Fpz→AFz-Fz | oPFC_to_dmPFC | | -0,3 | | -0.47 - -0.14 | | 0,043 | |
|  | Fpz→AF4-AF8-F4 | oPFC_to_RdlPFC | | -0,26 | | -0.39 - -0.13 | | 0,045 | |
|  | Fpz→F8-FC6 | oPFC_to_RIFG | | -0,28 | | -0.45 - -0.11 | | 0,095 | |
| **B. DTF statistics for incongruity resolution– MET vs ABS** | | | | | | | | | |
| **Direction of information flow (source_to_receiver)** | | | **Between-group Interaction effects** | | | | | |  |
| **Time window** | **Electrodes** | **ROI to ROI** | **Standardized Beta Coefficients** | | **Confidence Intervals** | | **Marginal R2** | |  |
| **0 – 1 s** |  | **Healthy controls > Schizophrenia outpatients** | | | | | | |  |
|  | T7→AF3-AF7-F3 | LaTL_to_LdlPFC | 0,27 | | 0.13 - 0.4 | | 0,069 | |  |
|  | F7-FC5→AFz-Fz | LIFG_to_dmPFC | 0,27 | | 0.16 - 0.38 | | 0,044 | |  |
|  | F7-FC5→Fp1 | LIFG_to_LvmPFC | 0,4 | | 0.23 - 0.58 | | 0,053 | |  |
|  | P3-P5→TP7-P7 | LIPL_to_LpTL | 0,2 | | 0.08 - 0.32 | | 0,023 | |  |
|  | TP7-P7→F7-FC5 | LpTL_to_LIFG | 0,19 | | 0.07 - 0.31 | | 0,02 | |  |
|  | P1-P2→AFz-Fz | Prec_to_dmPFC | 0,3 | | 0.19 - 0.4 | | 0,023 | |  |
|  | P1-P2→AF3-AF7-F3 | Prec_to_LdlPFC | 0,24 | | 0.15 - 0.32 | | 0,019 | |  |
|  | P1-P2→Fpz | Prec_to_oPFC | 0,29 | | 0.15 - 0.44 | | 0,042 | |  |
|  | P1-P2→AF4-AF8-F4 | Prec_to_RdlPFC | 0,23 | | 0.14 - 0.31 | | 0,026 | |  |
|  | P1-P2→F8-FC6 | Prec_to_RIFG | 0,19 | | 0.09 - 0.3 | | 0,028 | |  |
|  | P1-P2→TP8-P8 | Prec_to_RpTL | 0,16 | | 0.06 - 0.26 | | 0,028 | |  |
|  |  | **Healthy controls < Schizophrenia outpatients** | | | | | | |  |
|  | Fp1→P1-P2 | LvmPFC_to_Prec | -0,21 | | -0.33 - -0.08 | | NA | |  |
|  | Fpz→P3-P5 | oPFC_to_LIPL | -0,39 | | -0.55 - -0.23 | | 0,086 | |  |
|  | Fpz→P1-P2 | oPFC_to_Prec | -0,28 | | -0.4 - -0.15 | | 0,088 | |  |
|  | Fpz→CP6 | oPFC_to_RTPJ | -0,43 | | -0.66 - -0.2 | | NA | |  |
|  | T8→P3-P5 | RaTL_to_LIPL | -0,26 | | -0.41 - -0.1 | | 0,13 | |  |
|  | T8→P1-P2 | RaTL_to_Prec | -0,24 | | -0.37 - -0.11 | | 0,05 | |  |
|  | F8-FC6→AF3-AF7-F3 | RIFG_to_LdlPFC | -0,26 | | -0.36 - -0.16 | | 0,032 | |  |
|  | F8-FC6→F7-FC5 | RIFG_to_LIFG | -0,24 | | -0.37 - -0.12 | | 0,033 | |  |
|  | F8-FC6→Fp1 | RIFG_to_LvmPFC | -0,3 | | -0.47 - -0.13 | | 0,047 | |  |
|  | P4-P6→TP7-P7 | RIPL_to_LpTL | -0,28 | | -0.4 - -0.15 | | 0,039 | |  |
|  | P4-P6→CP5 | RIPL_to_LTPJ | -0,46 | | -0.62 - -0.29 | | 0,067 | |  |
|  | P4-P6→T8 | RIPL_to_RaTL | -0,29 | | -0.45 - -0.14 | | NA | |  |
|  | P4-P6→CP6 | RIPL_to_RTPJ | -0,35 | | -0.51 - -0.2 | | 0,071 | |  |
|  | TP8-P8→AFz-Fz | RpTL_to_dmPFC | -0,22 | | -0.34 - -0.1 | | 0,06 | |  |
|  | TP8-P8→AF3-AF7-F3 | RpTL_to_LdlPFC | -0,25 | | -0.35 - -0.15 | | 0,068 | |  |
|  | TP8-P8→F7-FC5 | RpTL_to_LIFG | -0,21 | | -0.33 - -0.08 | | 0,058 | |  |
|  | TP8-P8→AF4-AF8-F4 | RpTL_to_RdlPFC | -0,22 | | -0.32 - -0.12 | | 0,04 | |  |
|  | TP8-P8→F8-FC6 | RpTL_to_RIFG | -0,23 | | -0.36 - -0.11 | | 0,033 | |  |
|  | CP6→P3-P5 | RTPJ_to_LIPL | -0,28 | | -0.43 - -0.13 | | 0,039 | |  |
| **1 – 2 s** |  | **Healthy controls > Schizophrenia outpatients** | | | | | | |  |
|  | T7→AF3-AF7-F3 | LaTL_to_LdlPFC | 0,25 | | 0.11 - 0.38 | | 0,069 | |  |
|  | AF3-AF7-F3→P1-P2 | LdlPFC_to_Prec | 0,14 | | 0.06 - 0.21 | | 0,012 | |  |
|  | AF3-AF7-F3→P4-P6 | LdlPFC_to_RIPL | 0,19 | | 0.1 - 0.29 | | 0,016 | |  |
|  | TP7-P7→AFz-Fz | LpTL_to_dmPFC | 0,2 | | 0.09 - 0.31 | | 0,026 | |  |
|  | TP7-P7→AF3-AF7-F3 | LpTL_to_LdlPFC | 0,17 | | 0.08 - 0.26 | | 0,013 | |  |
|  | TP7-P7→F7-FC5 | LpTL_to_LIFG | 0,23 | | 0.12 - 0.35 | | 0,02 | |  |
|  | P1-P2→AFz-Fz | Prec_to_dmPFC | 0,17 | | 0.07 - 0.27 | | 0,023 | |  |
|  | P1-P2→P4-P6 | Prec_to_RIPL | 0,2 | | 0.1 - 0.29 | | 0,045 | |  |
|  | P1-P2→TP8-P8 | Prec_to_RpTL | 0,18 | | 0.08 - 0.28 | | 0,028 | |  |
|  |  | **Healthy controls < Schizophrenia outpatients** | | | | | | |  |
|  | T7→P4-P6 | LaTL_to_RIPL | -0,26 | | -0.42 - -0.11 | | 0,091 | |  |
|  | T8→P3-P5 | RaTL_to_LIPL | -0,42 | | -0.57 - -0.27 | | 0,13 | |  |
|  | P4-P6→P3-P5 | RIPL_to_LIPL | -0,28 | | -0.4 - -0.16 | | 0,029 | |  |
|  | P4-P6→TP7-P7 | RIPL_to_LpTL | -0,3 | | -0.42 - -0.18 | | 0,039 | |  |
|  | P4-P6→CP5 | RIPL_to_LTPJ | -0,34 | | -0.5 - -0.17 | | 0,067 | |  |
|  | P4-P6→P1-P2 | RIPL_to_Prec | -0,2 | | -0.29 - -0.1 | | 0,029 | |  |
|  | TP8-P8→AFz-Fz | RpTL_to_dmPFC | -0,34 | | -0.46 - -0.22 | | 0,06 | |  |
|  | TP8-P8→AF3-AF7-F3 | RpTL_to_LdlPFC | -0,27 | | -0.37 - -0.17 | | 0,068 | |  |
|  | TP8-P8→Fp1 | RpTL_to_LvmPFC | -0,3 | | -0.48 - -0.12 | | 0,062 | |  |
|  | TP8-P8→AF4-AF8-F4 | RpTL_to_RdlPFC | -0,32 | | -0.42 - -0.22 | | 0,04 | |  |
|  | TP8-P8→Fp2 | RpTL_to_RvmPFC | -0,28 | | -0.46 - -0.11 | | 0,035 | |  |
| **2 – 3 s** |  | **Healthy controls > Schizophrenia outpatients** | | | | | | |  |
|  | P3-P5→TP7-P7 | LIPL_to_LpTL | 0,23 | | 0.1 - 0.35 | | 0,023 | |  |
|  | P3-P5→P1-P2 | LIPL_to_Prec | 0,18 | | 0.08 - 0.28 | | 0,018 | |  |
|  | P1-P2→P4-P6 | Prec_to_RIPL | 0,2 | | 0.1 - 0.3 | | 0,045 | |  |
|  | P1-P2→TP8-P8 | Prec_to_RpTL | 0,21 | | 0.11 - 0.3 | | 0,028 | |  |
|  | T8→TP7-P7 | RaTL_to_LpTL | 0,28 | | 0.12 - 0.43 | | 0,083 | |  |
|  |  | **Healthy controls < Schizophrenia outpatients** | | | | | | |  |
|  | T7→P4-P6 | LaTL_to_RIPL | -0,27 | | -0.42 - -0.12 | | 0,091 | |  |
|  | T7→TP8-P8 | LaTL_to_RpTL | -0,4 | | -0.55 - -0.25 | | 0,125 | |  |
|  | Fp1→AFz-Fz | LvmPFC_to_dmPFC | -0,27 | | -0.43 - -0.11 | | 0,048 | |  |
|  | Fpz→P3-P5 | oPFC_to_LIPL | -0,31 | | -0.47 - -0.16 | | 0,086 | |  |
|  | Fpz→P1-P2 | oPFC_to_Prec | -0,22 | | -0.34 - -0.1 | | 0,088 | |  |
|  | Fpz→CP6 | oPFC_to_RTPJ | -0,44 | | -0.67 - -0.21 | | NA | |  |
|  | F8-FC6→AFz-Fz | RIFG_to_dmPFC | -0,25 | | -0.36 - -0.13 | | 0,03 | |  |
|  | P4-P6→CP5 | RIPL_to_LTPJ | -0,3 | | -0.46 - -0.14 | | 0,067 | |  |
|  | P4-P6→P1-P2 | RIPL_to_Prec | -0,18 | | -0.27 - -0.08 | | 0,029 | |  |
|  | P4-P6→CP6 | RIPL_to_RTPJ | -0,27 | | -0.43 - -0.12 | | 0,071 | |  |
|  | TP8-P8→AF4-AF8-F4 | RpTL_to_RdlPFC | -0,28 | | -0.38 - -0.18 | | 0,04 | |  |
| **C. DTF statistics for complete metaphor processing contrast – MET vs NEU** | | | | | | | | |  |
| **Direction of information flow (source_to_receiver)** | | | **Between-group Interaction effects** | | | | | |  |
| **Time window** | **Electrodes** | **ROI to ROI** | **Standardized Beta Coefficients** | | **Confidence Intervals** | | **Marginal R2** | |  |
| **0 – 1 s** |  | **Healthy controls > Schizophrenia outpatients** | | | | | | |  |
|  | AFz-Fz→AF3-AF7-F3 | dmPFC_to_LdlPFC | 0,23 | | 0.13 - 0.33 | | 0,054 | |  |
|  | AFz-Fz→Fp1 | dmPFC_to_LvmPFC | 0,32 | | 0.14 - 0.49 | | 0,095 | |  |
|  | F7-FC5→AFz-Fz | LIFG_to_dmPFC | 0,26 | | 0.14 - 0.37 | | 0,044 | |  |
|  | CP5→AFz-Fz | LTPJ_to_dmPFC | 0,31 | | 0.15 - 0.47 | | 0,112 | |  |
|  | CP5→AF4-AF8-F4 | LTPJ_to_RdlPFC | 0,36 | | 0.23 - 0.5 | | 0,065 | |  |
|  | CP5→F8-FC6 | LTPJ_to_RIFG | 0,35 | | 0.18 - 0.53 | | 0,06 | |  |
|  | CP5→Fp2 | LTPJ_to_RvmPFC | 0,41 | | 0.18 - 0.64 | | NA | |  |
|  | P4-P6→Fp1 | RIPL_to_LvmPFC | 0,29 | | 0.12 - 0.46 | | 0,028 | |  |
|  | CP6→T7 | RTPJ_to_LaTL | 0,41 | | 0.2 - 0.62 | | NA | |  |
|  |  | **Healthy controls < Schizophrenia outpatients** | | | | | | |  |
|  | T7→TP7-P7 | LaTL_to_LpTL | -0,29 | | -0.45 - -0.13 | | 0,038 | |  |
|  | T7→CP5 | LaTL_to_LTPJ | -0,36 | | -0.57 - -0.16 | | NA | |  |
|  | AF3-AF7-F3→P1-P2 | LdlPFC_to_Prec | -0,13 | | -0.2 - -0.05 | | 0,012 | |  |
|  | F7-FC5→P1-P2 | LIFG_to_Prec | -0,18 | | -0.28 - -0.09 | | 0,013 | |  |
|  | Fpz→T7 | oPFC_to_LaTL | -0,45 | | -0.67 - -0.22 | | NA | |  |
|  | Fpz→F7-FC5 | oPFC_to_LIFG | -0,38 | | -0.54 - -0.21 | | 0,064 | |  |
|  | Fpz→P3-P5 | oPFC_to_LIPL | -0,32 | | -0.48 - -0.16 | | 0,086 | |  |
|  | Fpz→P1-P2 | oPFC_to_Prec | -0,33 | | -0.46 - -0.21 | | 0,088 | |  |
|  | Fpz→P4-P6 | oPFC_to_RIPL | -0,43 | | -0.59 - -0.27 | | 0,096 | |  |
|  | Fpz→TP8-P8 | oPFC_to_RpTL | -0,37 | | -0.53 - -0.2 | | 0,084 | |  |
|  | Fpz→CP6 | oPFC_to_RTPJ | -0,54 | | -0.77 - -0.3 | | NA | |  |
|  | P4-P6→CP5 | RIPL_to_LTPJ | -0,27 | | -0.43 - -0.11 | | 0,067 | |  |
| **1 – 2 s** |  | **Healthy controls > Schizophrenia outpatients** | | | | | | |  |
|  | F7-FC5→TP8-P8 | LIFG_to_RpTL | 0,21 | | 0.1 - 0.33 | | 0,023 | |  |
|  | CP5→AFz-Fz | LTPJ_to_dmPFC | 0,47 | | 0.31 - 0.63 | | 0,112 | |  |
|  | CP5→AF4-AF8-F4 | LTPJ_to_RdlPFC | 0,29 | | 0.16 - 0.43 | | 0,065 | |  |
|  | F8-FC6→AFz-Fz | RIFG_to_dmPFC | 0,21 | | 0.09 - 0.32 | | 0,03 | |  |
|  | F8-FC6→P3-P5 | RIFG_to_LIPL | 0,2 | | 0.08 - 0.32 | | 0,049 | |  |
|  | F8-FC6→P1-P2 | RIFG_to_Prec | 0,16 | | 0.06 - 0.26 | | 0,03 | |  |
|  | F8-FC6→AF4-AF8-F4 | RIFG_to_RdlPFC | 0,2 | | 0.1 - 0.3 | | 0,027 | |  |
|  | F8-FC6→P4-P6 | RIFG_to_RIPL | 0,23 | | 0.11 - 0.35 | | 0,022 | |  |
|  | CP6→P1-P2 | RTPJ_to_Prec | 0,26 | | 0.14 - 0.37 | | 0,07 | |  |
|  | CP6→F8-FC6 | RTPJ_to_RIFG | 0,25 | | 0.1 - 0.41 | | 0,033 | |  |
|  | Fp2→P1-P2 | RvmPFC_to_Prec | 0,2 | | 0.09 - 0.32 | | NA | |  |
|  |  | **Healthy controls < Schizophrenia outpatients** | | | | | | |  |
|  | T7→TP7-P7 | LaTL_to_LpTL | -0,27 | | -0.42 - -0.11 | | 0,038 | |  |
|  | T7→CP5 | LaTL_to_LTPJ | -0,37 | | -0.57 - -0.17 | | NA | |  |
|  | Fpz→T7 | oPFC_to_LaTL | -0,41 | | -0.64 - -0.18 | | NA | |  |
|  | Fpz→P4-P6 | oPFC_to_RIPL | -0,29 | | -0.46 - -0.13 | | 0,096 | |  |
|  | Fpz→TP8-P8 | oPFC_to_RpTL | -0,45 | | -0.61 - -0.28 | | 0,084 | |  |
|  | Fpz→CP6 | oPFC_to_RTPJ | -0,41 | | -0.64 - -0.17 | | NA | |  |
|  | T8→TP8-P8 | RaTL_to_RpTL | -0,27 | | -0.43 - -0.11 | | 0,038 | |  |
|  | P4-P6→P3-P5 | RIPL_to_LIPL | -0,3 | | -0.42 - -0.18 | | 0,029 | |  |
|  | P4-P6→TP7-P7 | RIPL_to_LpTL | -0,28 | | -0.4 - -0.16 | | 0,039 | |  |
|  | P4-P6→P1-P2 | RIPL_to_Prec | -0,16 | | -0.26 - -0.07 | | 0,029 | |  |
|  | P4-P6→TP8-P8 | RIPL_to_RpTL | -0,19 | | -0.31 - -0.07 | | 0,062 | |  |
| **2 – 3 s** |  | **Healthy controls > Schizophrenia outpatients** | | | | | | |  |
|  | AFz-Fz→AF3-AF7-F3 | dmPFC_to_LdlPFC | 0,18 | | 0.08 - 0.28 | | 0,054 | |  |
|  | AFz-Fz→AF4-AF8-F4 | dmPFC_to_RdlPFC | 0,25 | | 0.14 - 0.35 | | 0,028 | |  |
|  | P1-P2→P4-P6 | Prec_to_RIPL | 0,17 | | 0.07 - 0.27 | | 0,045 | |  |
|  | P1-P2→TP8-P8 | Prec_to_RpTL | 0,19 | | 0.09 - 0.29 | | 0,028 | |  |
|  | T8→TP7-P7 | RaTL_to_LpTL | 0,31 | | 0.15 - 0.46 | | 0,083 | |  |
|  |  | **Healthy controls < Schizophrenia outpatients** | | | | | | |  |
|  | F7-FC5→P1-P2 | LIFG_to_Prec | -0,18 | | -0.27 - -0.08 | | 0,013 | |  |
|  | Fpz→P1-P2 | oPFC_to_Prec | -0,22 | | -0.34 - -0.09 | | 0,088 | |  |
|  | Fpz→F8-FC6 | oPFC_to_RIFG | -0,3 | | -0.47 - -0.14 | | 0,095 | |  |
|  | Fpz→P4-P6 | oPFC_to_RIPL | -0,33 | | -0.49 - -0.17 | | 0,096 | |  |
|  | Fpz→CP6 | oPFC_to_RTPJ | -0,44 | | -0.67 - -0.21 | | NA | |  |

List of Regions of Interest (ROIs) and directions of electrode pairs (source→receiver) revealed by analysis of three contrasts: A.) ABS vs NEU; B.) MET vs ABS; C.) MET vs NEU. Statistics presented as Standardized Beta Coefficients with Confidence Intervals in appropriate epochs for between-group differences (i.e. interaction group effects). Electrode - ROIs definition: Left (L); right (R); orbitofrontal cortex (oPFC – Fpz); ventromedial prefrontal cortex (vmPFC – Fp1/Fp2); dorsomedial prefrontal cortex (dmPFC – AFz-Fz); dorsolateral prefrontal cortex (dlPFC –AF3-AF7-F3/AF4-AF8-F4); inferior frontal gyrus (IFG – F7-FC5/F8-FC6); anterior temporal lobe (aTL – T7/T8); posterior temporal lobe (pTL – TP7-P7/TP8-P8); temporoparietal junction (TPJ – CP5/CP6); inferior parietal lobule (IPL – P3-P5/P4-P6); precuneus (Prec – P1-P2). NA – non available.
